# Spatiotemporal regulation of nervous system development in the annelid *Capitella teleta*

**DOI:** 10.1186/s13227-017-0076-8

**Published:** 2017-08-01

**Authors:** Abhinav Sur, Craig R. Magie, Elaine C. Seaver, Néva P. Meyer

**Affiliations:** 1 Biology Department, Clark University, 950 Main St., Worcester, MA 01610-1400 USA; 2 Department of Biological Sciences, Quinnipiac University, 275 Mount Carmel Ave., Hamden, CT 06518-1905 USA; 3 Whitney Laboratory for Marine Bioscience, 9505 Ocean Shore Blvd., St. Augustine, FL 32080-8610 USA

**Keywords:** Neurogenesis, Annelid, *Capitella teleta*, Spiralian, SoxB1, Neurogenin, Ash, NeuroD, Musashi, Prospero

## Abstract

**Background:**

How nervous systems evolved remains an unresolved question. Previous studies in vertebrates and arthropods revealed that homologous genes regulate important neurogenic processes such as cell proliferation and differentiation. However, the mechanisms through which such homologs regulate neurogenesis across different bilaterian clades are variable, making inferences about nervous system evolution difficult. A better understanding of neurogenesis in the third major bilaterian clade, Spiralia, would greatly contribute to our ability to deduce the ancestral mechanism of neurogenesis.

**Results:**

Using whole-mount in situ hybridization, we examined spatiotemporal gene expression for homologs of *soxB*, *musashi*, *prospero*, *achaete*–*scute*, *neurogenin*, and *neuroD* in embryos and larvae of the spiralian annelid *Capitella teleta*, which has a central nervous system (CNS) comprising a brain and ventral nerve cord. For all homologs examined, we found expression in the neuroectoderm and/or CNS during neurogenesis. Furthermore, the onset of expression and localization within the developing neural tissue for each of these genes indicates putative roles in separate phases of neurogenesis, e.g., in neural precursor cells (NPCs) versus in cells that have exited the cell cycle. *Ct*-*soxB1*, *Ct*-*soxB*, and *Ct*-*ngn* are the earliest genes expressed in surface cells in the anterior and ventral neuroectoderm, while *Ct*-*ash1* expression initiates slightly later in surface neuroectoderm. *Ct*-*pros* is expressed in single cells in neural and non-neural ectoderm, while *Ct*-*msi* and *Ct*-*neuroD* are localized to differentiating neural cells in the brain and ventral nerve cord.

**Conclusions:**

These results suggest that the genes investigated in this article are involved in a neurogenic gene regulatory network in *C. teleta*. We propose that Ct-SoxB1, Ct-SoxB, and Ct-Ngn are involved in maintaining NPCs in a proliferative state. Ct-Pros may function in division of NPCs, Ct-Ash1 may promote cell cycle exit and ingression of NPC daughter cells, and Ct-NeuroD and Ct-Msi may control neuronal differentiation. Our results support the idea of a common genetic toolkit driving neural development whose molecular architecture has been rearranged within and across clades during evolution. Future functional studies should help elucidate the role of these homologs during *C. teleta* neurogenesis and identify which aspects of bilaterian neurogenesis may have been ancestral or were derived within Spiralia.

**Electronic supplementary material:**

The online version of this article (doi:10.1186/s13227-017-0076-8) contains supplementary material, which is available to authorized users.

## Background

Neurogenesis refers to the process by which differentiated neurons are generated from neural precursor cells (NPCs), and a critical component of this process is to maintain a balance between cell proliferation and differentiation. Coordination between these two distinct processes is necessary for differential growth and generation of a variety of functional neural cell types at the correct time and place, and an imbalance can result in harmful developmental defects [1, 2]. Various signaling pathways, transcription factors, and RNA-binding proteins regulate neurogenesis, and alterations in neurogenic regulatory networks and patterns of gene expression underlie the evolution of many complex phenotypes, including nervous systems [3–6]. Therefore, studying gene expression of neurogenic factors across a broad range of taxa can help us understand evolution of the molecular mechanisms underlying nervous system development.

Molecular and cellular studies have revealed that vertebrates and insects share some neurogenic mechanisms but there are also differences [1, 7–10]. One commonality between arthropods and vertebrates is that dedicated NPCs generate the various neural cell subtypes by asymmetric cell division [7, 11]. Some of the key molecular regulators of neurogenesis include SoxB, basic helix-loop-helix (bHLH) group A transcription factors (including Achaete–Scute complex a or ASCa family members), and Notch signaling. In vertebrates, SoxB1 homologs (e.g., mouse Sox1, 2, and 3) are initially expressed throughout the neuroectoderm and then in mitotically active NPCs. SoxB1 expression is largely downregulated as NPCs begin to differentiate [12–16]. Vertebrate NPCs rely on SoxB1 transcription factors and Notch signaling to remain in a proliferative, undifferentiated state. In part, SoxB1 and Notch signaling maintain NPCs by reducing expression levels and activity of proneural bHLH transcription factors such as Neurogenin1 and 2 and Mash1 (an ASCa homolog). Proneural proteins in turn promote cell cycle exit and neuronal differentiation, repress SoxB1 activity, and upregulate expression of the Notch ligand *delta* [9, 12, 13, 17–21]. One mechanism by which proneural proteins suppress SoxB1 activity is by upregulating expression of the SoxB2 gene Sox21, which promotes neural differentiation [22].

The functions of the *D. melanogaster* SoxB homologs SoxNeuro and Dichaete (also known as Fish-hook) are similar to vertebrates in that they help maintain neuroblasts [20, 23]. SoxNeuro is expressed throughout the neuroectoderm but gets downregulated in delaminated neuroblasts [24–26], while Dichaete has a somewhat more dynamic expression pattern in the neuroectoderm and neuroblasts [27, 28]. Loss of function of SoxNeuro and Dichaete results in a loss of neuroblasts throughout the neuroectoderm and severe hypoplasia in the CNS [24, 26, 29]. Proneural bHLH factors in the ASCa family, particularly Achaete, Scute, and Lethal of Scute, are also involved in neurogenesis in insects. However, they have a slightly different function than in vertebrates—they promote fate specification of neuroblasts at the expense of epidermal stem cells. ASCa proteins upregulate *delta* expression in presumptive neuroblasts, and then Delta activates Notch on neighboring cells. Targets of activated Notch downregulate expression of *ASCa* genes, thus preventing cells from becoming neuroblasts [9, 30–32]. There is evidence that the SoxB proteins in *D. melanogaster* can directly regulate gene expression of *achaete* and *asense* [33–36]. However, it is not clear whether proneural bHLH proteins affect expression of *soxB* homologs as they do in vertebrates, and definitive SoxB2 homologs have not yet been identified in *D. melanogaster* [37, 38].

Differences in neurogenic mechanisms can also be seen within clades. For example, in earlier branching arthropods such as the spider *Cupiennius salei* and the myriapods *Glomeris marginata* and *Lithobius forficatus*, *ASCa* homologs (*ash*) are expressed in patches of mitotically quiescent neuroectodermal cells, which separate from the apical surface as groups and eventually differentiate into neural cells [39–43]. This contrasts with insects, where proneural gene expression initiates within each proneural cluster but then becomes limited to the neuroblast, which will go on to divide [44, 45]. Within select clades of Spiralia (e.g., Platyhelminthes, annelids, mollusks, and nemerteans), recent studies highlight a potential involvement of SoxB and proneural proteins during neurogenesis [46–49]. In the annelid *Platynereis dumerilii*, *Pdu*-*ASH* and *Pdu*-*Ngn* are expressed along the apical proliferating zone of the neuroectoderm, while *Pdu*-*SoxB* is expressed throughout the neuroectoderm at earlier stages [48, 49]. Such variation highlights the importance of studying neurogenesis in multiple species within clades in order to understand what aspects of bilaterian neurogenesis are ancestral and what aspects have been derived within particular taxa. Our understanding of neurogenesis in spiralians, including the molecular components, the exact role of each component, and the extent of variability in the molecular and cellular details of neurogenesis in this clade is still relatively incomplete. Furthermore, a proper understanding of neurogenesis in spiralians is required to reconstruct the evolution of nervous systems within Bilateria.

In this article, we extend previous studies to describe the spatiotemporal expression of candidate neurogenic genes in the annelid *Capitella teleta*. Some aspects of neurogenesis have previously been described in *C. teleta* [50]. However, neurogenic mechanisms underlying ventral nerve cord (VNC) development, including gene expression, have not yet been well characterized. We found that gene homologs of SoxB, Musashi (Msi), Prospero (Pros), Achaete–Scute (Ash), Neurogenin (Ngn), and NeuroD are expressed in developing neural tissue in *C. teleta*. The onset and duration of expression and spatial localization indicate roles during different phases of neurogenesis within the brain and VNC.

## Methods

### Animal care

*Capitella teleta* [51] adults were maintained in the laboratory as previously described [52, 53]. Animals were kept in bowls of artificial seawater (ASW) and mud at 19 °C. Every 2 weeks, the adult worms were transferred to new bowls in order to maintain the density of worms within each bowl. Broods were dissected using a clean pair of Dumont #5 forceps to release the different embryonic and larval stages reared by the females. Embryonic and larval stages were collected from different bowls and used for whole-mount in situ hybridization (WMISH) experiments.

### Isolation of *C. teleta* neurogenic gene homologs

Total RNA was extracted from mixed stage 1–9 embryos and larvae using the RNA Trizol extraction protocol (Molecular Research Center, Inc.) or the RNeasy Mini Kit (Qiagen). Reverse transcription reactions were conducted using the SMARTer RACE kit (Clontech). *Capitella teleta* homologs were identified by tBLASTn searches against the *C. teleta* genome and EST libraries (JGI, DOE). We identified two *soxB* orthologs and single orthologs of *musashi*, *prospero, neurogenin*, and *neuroD*. We named these genes *Ct*-*soxB1*, *Ct*-*soxB*, *Ct*-*msi*, *Ct*-*pros*, *Ct*-*ngn*, and *Ct*-*neuroD*, respectively. Fragments of the coding sequences of these genes were amplified by PCR using gene-specific primers as follows.

*Ct*-*soxB1* (GenBank accession # MF508645): 917 bp containing some 5′ UTR, an HMG box, and a partial Soxp domain; Fwd primer 5′-CAAAGTCCTCGCTCAAAGCAG and Rev primer 5′-GCATGTATCCGTTCATGTTCATAGAG.

*Ct*-*soxB* (MF508646): 740 bp containing some 5′ UTR, an HMG box, and a partial Soxp domain; Fwd primer 5′-TTACCCTTCAACAAATCTAACTGC and Rev primer 5′-CGTATGGCGAGTAGAAAGCTC.

*Ct*-*msi* (MF508642): 729 bp containing a majority of the coding domain, including both RNA recognition motifs (RRMs), and some 3′-UTR; Fwd primer 5′-AGCCAGCAATCTACGTCAGG; Rev primer 5′-CACCACAGCAACGTGTTACC.

*Ct*-*prospero* (MF508647): 711 bp containing some 5′ UTR, the open reading frame with the entire homeo-prospero domain (HPD), and some 3′-UTR; Fwd primer 5′-CCAAGAACAGAAAAAGCAC; Rev primer 5′-TGTTTTGACTGCTTGATA.

*Ct*-*neurogenin* (MF508643): 778 bp of open reading frame with the entire bHLH domain; Fwd primer 5′-GGTCAATCTGACAGCAAGCA; Rev primer 5′-GGTAATGTCCTTGGTAACCTGGC.

*Ct*-*neuroD* (MF508644): 669 bp of open reading frame with the entire neuronal bHLH domain; Fwd primer 5′-ATGGCTAAAGCAGGAGATG; Rev primer 5′-TTCGGGTGATAGCGAGTAGG.

PCR products were TA cloned into the pGEM-T Easy vector (Promega) and sequenced. These gene fragments were used as templates to generate DIG-labeled anti-sense RNA probes for WMISH. A fragment of *Ct*-*ash1* was previously clones and is described in [50].

### Gene orthology analyses

Amino acid sequences for Sox family proteins (Additional file 1: Figure S1) and Musashi family proteins (Additional file 2: Figure S2) were aligned in MacVector 15.1 (MacVector, Inc.) via the T-Coffee alignment tool. The alignments were trimmed using trimAl v1.2b [54] via the gappyout algorithm. The best-fit model of protein evolution for each alignment was determined via ProtTest v3.2 [55, 56]. Bayesian phylogenetic analyses were performed on these alignments with MrBayes v3.2 [57] using the LG+G (Sox) or LG+G+I (Musashi) models of protein evolution [58] with 1,000,000 generations sampled every 100 generations and four chains. Fifty percent majority-rule consensus trees were produced from the last 7500 trees of each analysis representing 750,000 stationary generations. Consensus trees were visualized with FigTree v1.4.3 [59]. Posterior probabilities and branch lengths were calculated from these consensus trees. Maximum likelihood phylogenetic analyses were performed on these alignments with RAxML v8.2.10 [60] using the LG+G model of protein evolution and rapid bootstrapping with 500 replicates. Consensus trees were produced using the web-based interactive tree of life (iTOL) v3.5 [61]. Branches with a bootstrap value below 45 were collapsed.

### In situ hybridization

Multiple animals of each stage were obtained from different broods and treated for fixation as described elsewhere [50, 52]. All fixations for WMISH were done in 4% paraformaldehyde in ASW for more than 6 h. After fixation, animals were serially dehydrated in methanol and stored at −20 °C. WMISH was conducted as described previously [62]. Briefly, animals were hybridized for a minimum of 72 h at 65 °C with 1 ng/µl of each probe. DIG-labeled RNA probes were generated using the MegaScript SP6 or T7 transcription kit (ThermoFisher Scientific). Spatiotemporal RNA localization was observed using an NBT/BCIP color reaction. After WMISH, animals were stained with 0.1 µg/ml Hoechst 33342, cleared in 80% glycerol in PBS, and mounted on microscope slides for DIC imaging.

### Microscopy

Images were taken using DIC optics on either a (1) Zeiss M2 microscope with an 18.0 megapixel EOS Rebel T2i digital camera (Canon) or (2) a Zeiss Axioskop 2 microscope and a SPOT Flex digital camera (Diagnostic Instruments, Inc, MI, USA) in conjunction with the Spot Advanced version 4.6 software (Diagnostic Instruments, Inc). Animals from multiple broods were imaged for each developmental stage and gene. WMISH images were cropped and brightness, contrast, and color balance edited to maintain consistency within plates using Adobe Photoshop CC. Figure panels were constructed using Adobe Illustrator CC.

## Results

### Overview of larval neurogenesis in *Capitella teleta*

**Fig. 1:**
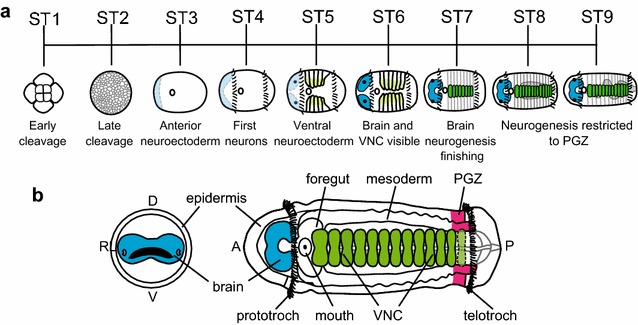
Overview of *Capitella teleta* neural development. **a** Developmental staging chart modified from [52, 53]. The stage 3 animal depicted has a mouth opening. Landmarks of CNS development are indicated. Each stage lasts ~1 day at 19 °C. The anterior neuroectoderm is indicated in *light blue* and the brain is indicated in *blue*. The ventral neuroectoderm is indicated in *light green* and the VNC in *green*. **b** Anterior (*left*) and ventral (*right*) diagrams modified from [52] of a stage 6 larva with different tissues indicated. The brain is *blue*, the VNC is *green*, and the PGZ is *pink*. *A* anterior, *D* dorsal, *L* left, *P* posterior, *PGZ* posterior growth zone, *R* right, *V* ventral

*Capitella teleta* development takes approximately 9 days at 19 °C to develop from a fertilized oocyte (stage 0) to a larva that is competent to metamorphose (stage 9), and an embryonic and larval staging system based on morphological features has previously been described (Fig. 1a) [53]. Further subdivision of stage 3 based on progression through gastrulation (epiboly, blastopore formation, blastopore closing, and mouth formation) is described in [50]. *Capitella teleta* has a non-feeding, swimming larva with an anterior brain (Fig. 1b, blue) and a VNC (Fig. 1b, green). Based on a previous study [50], the first sign of neurogenesis in the brain occurs early during gastrulation, when a region of anterior ectoderm (referred to as the anterior neuroectoderm or anterior ectodermal thickening) becomes thickened relative to the surrounding cells (Fig. 1a, ST3 light blue). Cells within this thickened region generate the brain through cell division and ingression. As cells ingress, two distinct spatial subregions in the developing brain are identifiable—a surface/apical region with actively dividing cells and an internal/basal region with cells that have exited the cell cycle and exhibit signs of neuronal differentiation [50, 52]. The first differentiated neurons are visible in the brain at early larval stages (stage 4) followed shortly thereafter by neurons in the VNC at stage 5 [50, 52]. Based on DiI labeling and cell division patterns, neurogenesis occurs in the episphere from stages 3–7 [50].

The VNC in *C. teleta* develops from anterior to posterior, and cell division occurs throughout the ventral neuroectoderm from stages 4–6 [53]. Toward the end of stage 6, cell division begins to be restricted to the posterior end of the larva and by stage 7 largely takes place in the posterior growth zone (PGZ; Fig. 1b, pink) [53]. Furthermore, by stage 7, mature neurons are present in all 13 ganglia formed in larvae as demonstrated by expression of the pan-neuronal marker *Ct*-*syt1* and the presence of neurons with FMRF-like immunoreactivity [50, 52]. By stage 8, cell division in the trunk has subsided except in the PGZ [53], suggesting that neurogenesis in the first 13 ganglia is complete. Ganglia continue to be added to the VNC from the PGZ as new segments form in juveniles and adults [53].

### *Ct*-*soxB1* and *Ct*-*soxB* expression

**Fig. 2:**
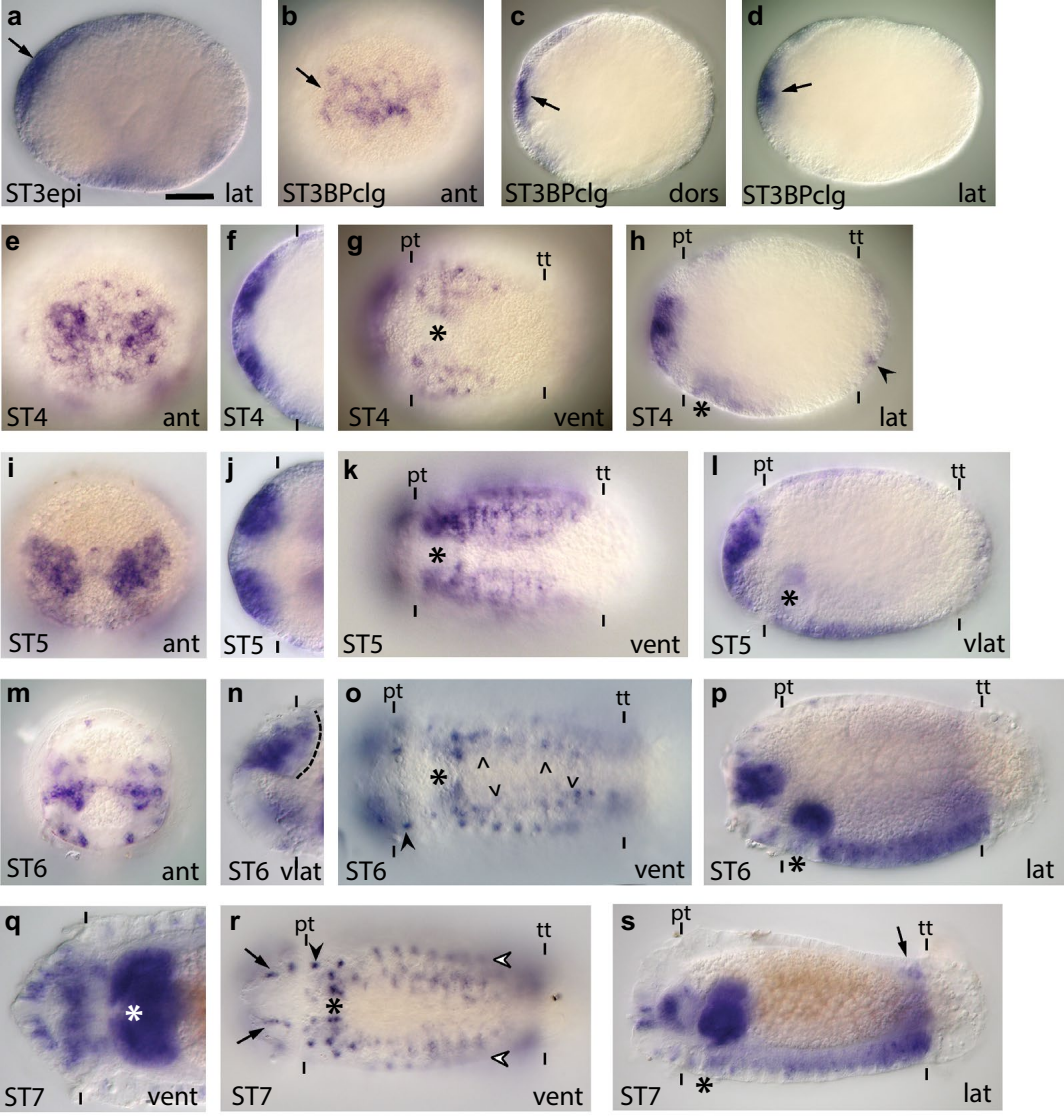
*Ct*-*soxB1* is expressed in neuroectoderm and the developing CNS. **a**–**s** *Ct*-*soxB1* transcripts were detected in stages 3–7 animals using whole-mount in situ hybridization (WMISH). The *scale bar* in A is 40 µm; all other images are to the same scale unless otherwise noted. *Arrows* in (**a**–**d**) denote expression in the anterior neuroectoderm. *Black arrowhead* in (**h**) points to expression in the pygidial ectoderm. *Dashed line* in (**n**) indicates the basal boundary of the brain lobe. *Arrowheads* in (**o**) denote *Ct*-*soxB1* ^+^ cells in the VNC. *Black arrowheads* in (**o**) and (**r**) point to a circumferential row of *Ct*-*soxB1* ^+^ cells immediately posterior to the prototroch. *White arrowheads* in (**r**) show ventrolateral rows of *Ct*-*soxB1* ^+^ cells on either side of the developing VNC. *Arrows* in (**r**) indicate ventral ectodermal *Ct*-*soxB1* ^+^ cells in the head. *Arrow* in (**s**) denotes expression in the PGZ. In each panel, the stage of the animal is indicated in the *lower left*, and the view is indicated in the *lower right* (*lat* lateral, *vlat* ventrolateral, *vent* ventral, *ant* anterior). In all lateral and ventrolateral views, anterior is to the left and ventral down; in all ventral views, anterior is to the left; and in all anterior views, ventral is down. Panels (**f**) and (**j**) are cropped images of the episphere from a ventral view. Panel (**n**) is a cropped image of the episphere from a ventrolateral view. Panel (**q**) is a cropped image of the episphere at 2.1× magnification. An *asterisk* marks the position of the mouth, and the prototroch (pt) and telotroch (tt) are labeled with *dashes*. In (**f**), (**j**), (**n**), and (**q**), the prototroch is denoted with *dashes*. ST3epi, stage 3 epiboly; STBPclg, stage 3 blastopore closing

**Fig. 3:**
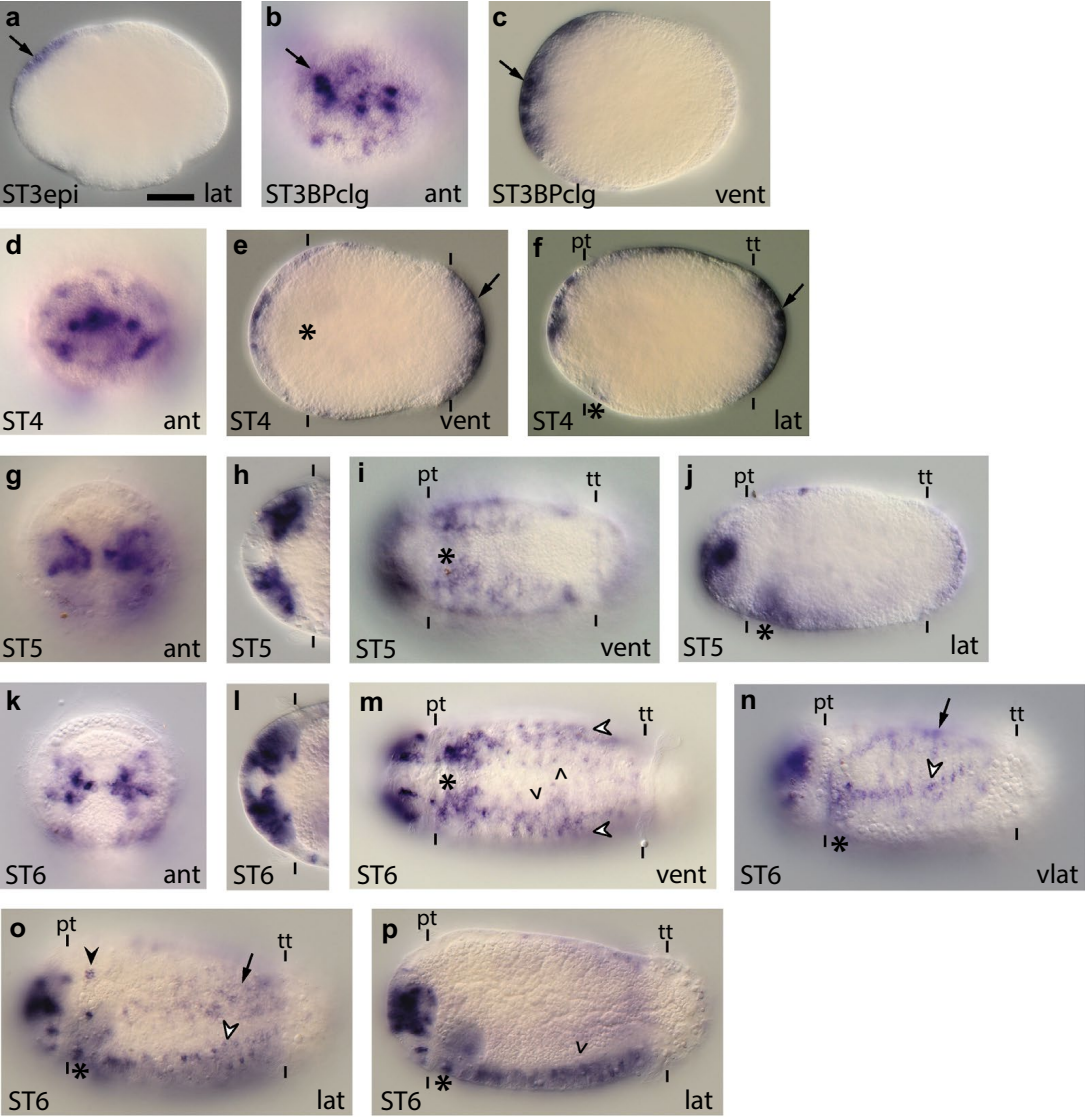
*Ct*-*soxB* is expressed in regions overlapping *Ct*-*soxB1* expression. **a**–**p** *Ct*-*soxB* transcripts were detected in stage 3–6 animals using WMISH. The *scale bar* in (**a**) is 40 µm; all other images are to the same scale unless otherwise noted. *Arrows* in (**a**–**c**) indicate expression in the anterior neuroectoderm. *Arrows* in (**e**) and (**f**) point to pygidial expression. *Arrowheads* in (**m**) and (**p**) point to *Ct*-*soxB* ^+^ cells in the VNC. *White arrowheads* in (**m**)–(**o**) point to ventrolateral rows of *Ct*-*soxB* ^+^ cells in the ectoderm. *Arrows* in (**n**) and (**o**) indicate lateral ectodermal patches of *Ct*-*soxB1* expression. *Black arrowhead* in (**o**) denotes a circumferential ring of cells posterior to the prototroch. In each panel, the stage of the animal is indicated in the *lower left*, and the view is indicated in the *lower right* (*lat* lateral, *vlat* ventrolateral, *vent* ventral, *ant* anterior). In all lateral and ventrolateral views, anterior is to the left and ventral down; in all ventral views, anterior is to the left; and in all anterior views, ventral is down. Panels (**h**) and (**l**) are cropped images of the episphere from a ventral view. An *asterisk* marks the position of the mouth, and the prototroch (pt) and telotroch (tt) are labeled with *dashes*. In (**h**) and (**l**), the prototroch is denoted using *dashes*. ST3epi, stage 3 epiboly; STBPclg, stage 3 blastopore closing

The Sox proteins are subdivided into groups A–G based on their HMG-box sequences [63–65]. The group B proteins have been further subdivided into groups B1 and B2 based on sequence differences outside the HMG-box domain [37, 66, 67], and the ability of some SoxB1 proteins to act as transcriptional activators and some SoxB2 proteins to act as repressors. However, clear phylogenetic support for a distinct SoxB2 clade using the HMG box has not been found [65, 68–70]. We screened the *C. teleta* genome and identified multiple genes with a Sox-like HMG box, and based on phylogenetic analyses, *C. teleta* has single orthologs of SoxA, C, D, E, F, and G and two SoxB orthologs (Additional file 3: Figure S3). Bayesian analysis found strong support and maximum likelihood analysis found moderate support for a SoxB1 clade nested within a larger SoxB clade that contained the vertebrate SoxB2 proteins. One *C. teleta* Sox gene grouped within the SoxB1 clade and one within the larger SoxB clade (Additional file 3: Figure S3). Similar to other analyses, we did not identify a clear SoxB2 clade, so we named the two SoxB orthologs Ct-SoxB1 and Ct-SoxB. As described below, the two *soxB* orthologs have similar, but distinct expression patterns in *C. teleta* (Figs. 2, 3; Additional file 4: Figure S4).

Description of *Ct*-*soxB1*: *Ct*-*soxB1* transcript was detected in zygotes and early cleavage stages (two cells through late cleavage; data not shown). Once the embryos begin to gastrulate (stage 3), *Ct*-*soxB1* is expressed in the anterior ectodermal thickening (Fig. 2a–d, arrow), which comprises brain NPCs [50]. *Ct*-*soxB1* is also present at lower levels in a majority of micromeres during gastrulation (data not shown), but becomes downregulated in most cells outside of the anterior ectoderm by the end of stage 3 (Fig. 2b–d). After gastrulation is complete (stage 4), *Ct*-*soxB1* continues to be expressed throughout the anterior ectoderm, but is more highly expressed in the neuroectoderm (Fig. 2e, f, h). At stage 4, *Ct*-*soxB1* transcript also is detected in the trunk in the presumptive neuroectoderm of the VNC (ventral neuroectoderm; Fig. 2g) and in patches of cells dispersed throughout the pygidial ectoderm (Fig. 2h, black arrowhead points to one cell). Furthermore, the onset of *Ct*-*soxB1* expression in the ventral neuroectoderm precedes formation of ganglia and expression of neural differentiation markers such as *Ct*-*syt1* by at least one full stage, or 1 day at 19 °C [52].

During stage 5, *Ct*-*soxB1* is expressed in surface neuroectoderm, where cells are dividing, as well as in the majority of cells that have already internalized to form the brain (Fig. 2i, j, l). In the trunk, the expression of *Ct*-*soxB1* expression expands considerably, extending from the mouth to the telotroch (Fig. 2k) and covering the entire ventrolateral domain (data not shown). *Ct*-*soxB1* is still expressed in the pygidial ectoderm, as well as in two new domains, in the foregut (Fig. 2k, l) and dorsolateral ectoderm (data not shown). At stage 6, *Ct*-*soxB1* continues to be expressed in the surface neuroectoderm in the episphere as well as in cells in the developing brain (Fig. 2m, n, p), although expression appears to be excluded from the most basal portion of the brain (Fig. 2n, dashed line demarcates the basal edge of the brain). *Ct*-*soxB1* is also present in individual ectodermal cells that form a circumferential ring immediately posterior to the prototroch (Fig. 2o, black arrowhead). Within the trunk at stage 6, a subset of ventral neuroectodermal cells express *Ct*-*soxB1* as well as individual cells within the developing VNC (Fig. 2o, arrowheads; Fig. 2p). At this stage, lower levels of *Ct*-*soxB1* expression are still detected in the developing foregut and the lateral and dorsolateral ectoderm (Fig. 2o, p and data not shown).

By stage 7, as larval neurogenesis in the episphere is nearing completion, *Ct*-*soxB1* is expressed in the brain and the overlying epidermis (Fig. 2q, s). There also is a new region of single cells in the ventral epidermis in the episphere (Fig. 2r, arrows) and adjacent to the opening of the mouth (Fig. 2r). Within the trunk, *Ct*-*soxB1* continues to be expressed in a circumferential row of epidermal cells posterior to the prototroch (Fig. 2r, black arrowhead). *Ct*-*soxB1* is expressed in a small subset of cells in the VNC as well as in six longitudinal rows of ectodermal cells (three rows on either side of the body), which are likely part of the peripheral nervous system. The ventral-most pair of longitudinal rows is positioned on either side of the VNC (Fig. 2r, white arrowheads). The second pair of longitudinal rows is also ventrolateral, while the third pair of rows is dorsolateral (data not shown). Trunk expression also persists in the foregut (Fig. 2s) and can be seen in the PGZ, where new segments and ganglia of the VNC are being added (Fig. 2s, arrow). The pattern of expression at stage 8 is very similar to that observed at stage 7 (Additional file 4: Figure S4a–c; arrowhead in a points to expression the VNC; arrow in c points to expression in the epidermis in the episphere). Moreover, the *Ct*-*soxB1* ^+^ cells in the ventral epidermis are quite distinct at stage 8, forming a ‘grid-like’ pattern (Additional file 4: Figure S4a). By stage 9, *Ct*-*soxB1* is downregulated in most of the larva except for a few cells in the epidermis of the episphere and in the PGZ (Additional file 4: Figure S4d).

Description of *Ct*-*soxB*: *Ct*-*soxB* was detected in zygotes and early cleavage stage embryos (two cells through birth of the third quartet of micromeres; data not shown). Once gastrulation begins, *Ct*-*soxB* is expressed in the anterior ectodermal thickening (Fig. 3a, arrow). In later stage 3 embryos, *Ct*-*soxB* is expressed in patches of cells throughout the anterior ectoderm, with higher levels in the anterior ectodermal thickening (Fig. 3b, c, arrow), which is similar to *Ct*-*soxB1* at this stage. *Ct*-*soxB* is also expressed in a circumferential band of cells in the anterior half of the trunk (data not shown). During stage 4, *Ct*-*soxB* continues to be expressed in the anterior ectoderm, and expression in the neuroectoderm appears to be confined to surface cells (Fig. 3d–f). By the end of stage 4, *Ct*-*soxB* expression also becomes visible in cells around the stomodeum (data not shown) and in the pygidial ectoderm, posterior to the telotroch, (Fig. 3e, f, arrows). Unlike *Ct*-*soxB1* at stage 4, Ct-soxB does not appear to be expressed in the ventral neuroectoderm at this stage.

During stage 5, as the brain lobes become more visible, *Ct*-*soxB* continues to be expressed in surface anterior neuroectoderm and also begins to be expressed and at varying intensities throughout the developing brain (Fig. 3g, h, j). In the trunk, *Ct*-*soxB* is expressed around the stomodeum and in patches within the ventral neuroectoderm (Fig. 3i). Pygidial expression continued to be detected at stage 5 (Fig. 3j), but not thereafter. At stage 6, *Ct*-*soxB* continues to be expressed in patches of cells in surface anterior neuroectoderm and in the developing brain (Fig. 3k, l, p). Expression in the trunk at stage 6 encompasses cells around the stomodeum and a small subset of cells in the VNC (Fig. 3m, p, arrowheads). In addition, *Ct*-*soxB* is expressed in two ventrolateral rows of cells on either side of the VNC (Fig. 3m–o white arrowheads) and in patches of cells laterally (Fig. 3n, o, arrows) and dorsolaterally (data not shown). There is also a circumferential row of *Ct*-*soxB* ^+^ cells immediately posterior to the prototroch (Fig. 3o, black arrowhead).

At stage 7, *Ct*-*soxB* is expressed in a ‘grid-like’ pattern in the ventral epidermis of the episphere and around the mouth opening (Additional file 4: Figure S4e), similar to *Ct*-*soxB1*. Additional expression domains include the brain lobes, the epidermis overlying the brain, and a subset of cells in the pharynx (Additional file 4: Figure S4f). *Ct*-*soxB* was also detected in a few cells in the developing VNC (Additional file 4: Figure S4e, arrowheads) as well as in the ventrolateral ectoderm (data not shown). *Ct*-*soxB* expression at stage 8 is similar to that at stage 7, except that expression in the VNC and ventrolateral ectoderm was not detected (Additional file 4: Figure S4g). By stage 9, expression was only detected in the brain (Additional file 4: Figure S4h).

### *Ct*-*msi* homolog expression

**Fig. 4:**
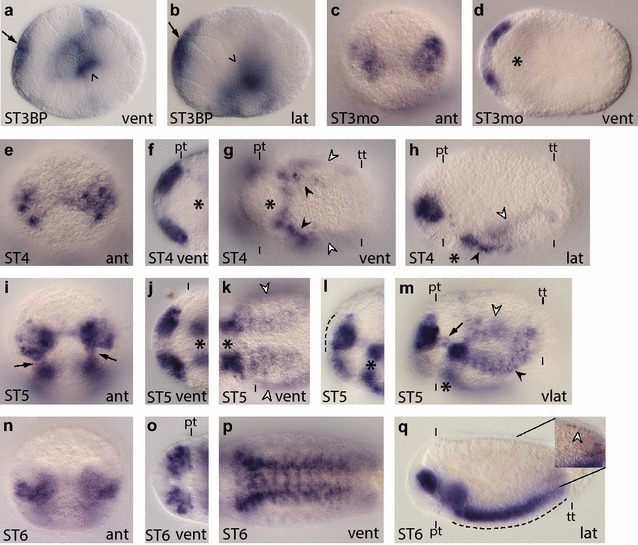
*Ct*-*msi* is broadly expressed in the developing brain and VNC. **a**–**q** *Ct*-*msi* transcripts were detected across stages 3–6 using WMISH. Images are to the same scale as in Figs. 2 and 3. *Arrows* in panels (**a**) and (**b**) indicate *Ct*-*msi* expression in the anterior neuroectoderm, while *arrowheads* indicate *Ct*-*msi* ^+^ cells around the blastopore. *White arrowheads* in panels (**g**), (**h**), (**k**), and (**m**) indicate expression in the mesoderm. *Black arrowheads* in (**m**) and (**h**) indicate expression in the presumptive VNC. *Arrows* in (**i**) and (**m**) indicate expression near the circumesophageal connectives. The *white arrowhead* in the *inset* in (**q**) indicates a longitudinal row of *Ct*-*msi* ^+^ cells positioned between the mesoderm and endoderm. In each panel, the stage of the animal is indicated in the *lower left*, and the view is indicated in the *lower right* (*lat* lateral, *vlat* ventrolateral, *vent* ventral, *ant* anterior). In all lateral and ventrolateral views, anterior is to the left and ventral down; in all ventral views, anterior is to the left; and in all anterior views, ventral is down. Panels (**f**), (**j**), and (**o**) are cropped images of the episphere from a ventral view. Panel (**l**) is a cropped image of the episphere from a ventrolateral view. Panels (**k**) and (**p**) are cropped images of the trunk. An *asterisk* marks the position of the mouth, and the prototroch (pt) and telotroch (tt) are labeled with *dashes*. In (**j**) the prototroch is denoted with a *dash*. STBP, stage 3 blastopore; ST3mo, stage 3 mouth

Musashi (Msi) homologs are RNA-binding proteins, some of which are expressed in the nervous system in several bilaterian taxa and have functions ranging from NPC maintenance, promotion of cell division, regulation of asymmetric cell division, and neuronal differentiation [71–77]. Vertebrates have two paralogs, Msi1 and Msi2 [76, 78]. The original *musashi* gene was cloned in *D. melanogaster* [75, 79, 80], but this gene appears to be an insect-specific paralog of RBP6, which is more closely related to the vertebrate paralogs, Msi1 and Msi2 [81]. In *C. teleta*, we identified one Msi ortholog, Ct-Msi, which groups with the vertebrate Msi1 and 2 homologs and the insect RBP6 homologs with strong support (Additional file 5: Figure S5). We also identified three additional proteins in the *C. teleta* genome that have similar RNA-binding domains. One protein grouped within the Dazap clade (Ct-Dazap, protein ID number 219071, scaffold 154). The two other proteins (protein ID numbers 175465 and 169740, on scaffolds 164 and 32, respectively) clustered with two predicted proteins from *Lottia gigantea* that are next to each other on scaffold 61 in the *L. gigantea* genome. These four proteins formed a sister clade to the heterogeneous nuclear ribonuclear protein D (HNRPD) clade (Additional file 5: Figure S5). We describe expression for *Ct*-*msi* during embryonic and larval stages (Fig. 4; Additional file 6: Figure S6a–e).

*Ct*-*msi* transcripts were detected in zygotes and two-cell embryos through birth of the third quartet of micromeres, although expression was only detected in a few micromeres after birth of the third quartet (data not shown). During gastrulation, *Ct*-*msi* was detected in the anterior ectodermal thickening (Fig. 4a, b, arrow) as well as around the blastopore opening and in internalized endodermal cells (Fig. 4a, b, arrowhead). From late stage 3 through 4, *Ct*-*msi* is expressed in the anterior neuroectoderm (Fig. 4c–f, h), in cells abutting the stomodeum, and in the mesodermal bands (Fig. 4g, h; white arrowheads point to mesodermal staining). Two longitudinal rows of expression that may coincide with the developing VNC at this stage were also detected (Fig. 4g, h, black arrowheads).

*Ct*-*msi* continues to be expressed in the developing brain at stage 5 (Fig. 4i, j, l), and expression appears to be excluded from surface neuroectoderm (Fig. 4l, dashed line denotes the apical surface of the ectoderm). Similar to brain expression at this stage, *Ct*-*msi* is expressed in the developing VNC (Fig. 4k, m; black arrowheads in m point to expression in the VNC) and appears to be subsurface, although it is more difficult to discern surface versus subsurface for the VNC at this stage. Expression in the mesodermal bands (Fig. 4k, l, white arrowheads) and foregut (Fig. 4k–m) continues to be present at stage 5. *Ct*-*msi* also begins to be expressed in cells associated with the circumesophageal connectives (Fig. 4i, m arrows). At stage 6, *Ct*-*msi* expression persists throughout the developing brain (Fig. 4n, o, q) but is not present in the overlying ectoderm (Fig. 4q). Within the trunk, *Ct*-*msi* is expressed in the foregut and throughout the VNC (Fig. 4p, q). Interestingly, *Ct*-*msi* does not appear to be expressed in the surface ventral neuroectoderm (Fig. 4q, dashed line denotes the apical surface of the ectoderm). Expression in the mesoderm is greatly decreased at stage 6, although we did detect posteriorly localized, longitudinal rows of *Ct*-*msi* ^+^ cells (at least three rows on each side of the larva) positioned between the mesoderm and endoderm. These cells are positioned laterally and dorsally, and one dorsally localized row is indicated with a white arrowhead in the inset in Fig. 4q. At stages 7–9, *Ct*-*msi* continues to be expressed in the brain and VNC, but is not expressed in the mesoderm or surface ectoderm (Additional file 6: Figure S6a–e). We also did not detect *Ct*-*msi* transcript in the PGZ (Additional file 6: Figure S6a, b, d, e), where the majority of cell division occurs after stage 7 [53]. Once the foregut has differentiated into a pharynx and esophagus (stage 8), it is clear that *Ct*-*msi* expression is restricted to the pharynx (Additional file 6: Figure S6d).

### *Ct*-*pros* homolog expression

**Fig. 5:**
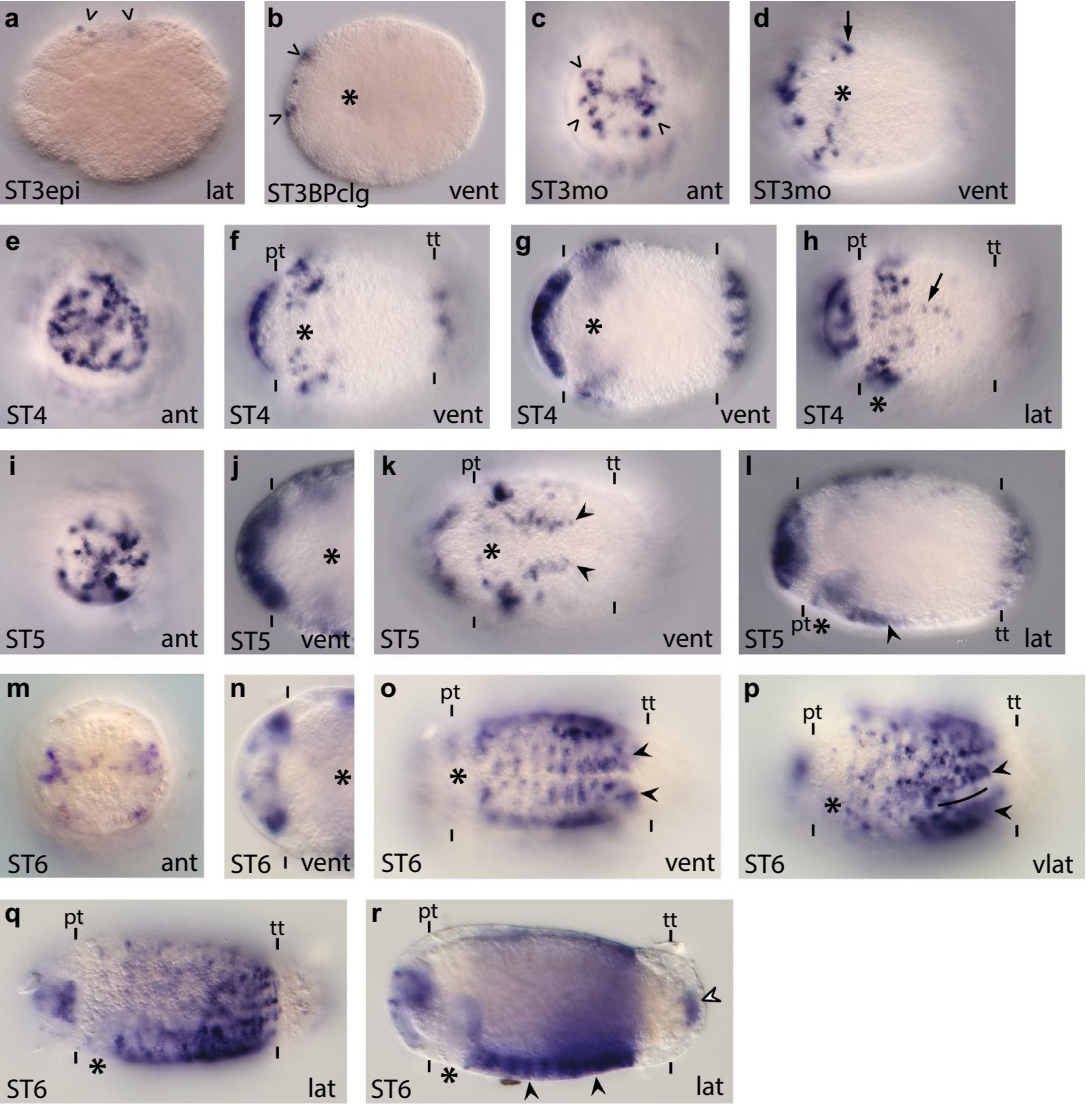
*Ct*-*pros* is expressed in single cells throughout the ectoderm and developing CNS. **a**–**r** *Ct*-*pros* transcripts were detected in stage 3–6 animals using WMISH. Images are to the same scale as in Figs. 2 and 3. *Arrowheads* in (**a**) indicate *Ct*-*pros* ^+^ cells in the animal pole. *Arrowheads* in (**b**) and (**c**) indicate surface *Ct*-*pros* ^+^ cells within the anterior ectoderm. The *arrow* in (**d**) points to expression in single ectodermal cells in the peristomium. The *arrow* in (**h**) denotes a few lateral *Ct*-*pros* ^+^ ectodermal cells just posterior to the peristomium. *Black arrowheads* in panels (**k**), (**l**), (**o**), (**p**), and (**r**) point to expression in the developing VNC. The *line* in panel (**p**) denotes the ventral midline. The *white arrowhead* in (**r**) points to expression in the rectum. In each panel, the stage of the animal is indicated in the *lower left*, and the view is indicated in the *lower right* (*lat* lateral, *vlat* ventrolateral, *vent* ventral, *ant* anterior). In all lateral and ventrolateral views, anterior is to the left and ventral down; in all ventral views, anterior is to the left; and in all anterior views, ventral is down. Panels (**j**) and (**n**) are cropped images of the *head*. An *asterisk* marks the position of the mouth, and the prototroch (pt) and telotroch (tt) are labeled with *dashes*. In panel G, *dashes* mark prototroch and telotroch, respectively, from left to right. ST3epi, stage 3 epiboly; STBPclg, stage 3 blastopore closing; ST3mo, stage 3 mouth

Prospero (Pros or Prox) homologs are a family of homeobox genes that are characterized by the presence of a C-terminal homeodomain followed by a unique Prospero domain [82]. Where examined, Prospero homologs seem to play a role in asymmetric cell division and differentiation [49, 83–89]. A screen of the *C. teleta* genome yielded one *prospero* homolog, *Ct*-*pros*, which encodes a protein with a homeodomain and a Prospero domain. *Ct*-*Pros* was previously found to cluster with *P. dumerilii* Prox and *D. melanogaster* Prospero [49]. *Ct*-*pros* transcripts were detected from cleavage stages (data not shown) through stage 9 (Fig. 5; Additional file 6: Figure S6f–j).

*Ct*-*pros* transcripts were detected in zygotes and in early cleavage stage embryos (two cells though birth of the third quartet of micromeres; data not shown). During early epiboly, *Ct*-*pros* is expressed in 3–4 cells at the animal pole (Fig. 5a, arrowheads). As the blastopore closes, a few *Ct*-*pros* ^+^ cells can be detected within the anterior neuroectoderm (Fig. 5b, arrowheads), in the region where the mouth will form, and in lateral and dorsal ectoderm (data not shown). In late stage 3 embryos, *Ct*-*pros* is expressed in single cells in the anterior ectoderm, including in the thickened region that will generate the brain (Fig. 5c, arrowheads). In the trunk, a circumferential ring of *Ct*-*pros* ^+^ cells can be detected in the ectoderm of the peristomium, at the anterior–posterior position of the mouth (Fig. 5d, arrow). Within the peristomium, there appear to be fewer *Ct*-*pros* ^+^ cells at the dorsal midline (data not shown). Finally, *Ct*-*pros* is expressed in single cells throughout the pygidial ectoderm (data not shown). At stage 4, *Ct*-*pros* is expressed in more cells in the anterior ectoderm and in subsurface cells in the developing brain (Fig. 5e, g). Expression of *Ct*-*pros* in the peristomial ectoderm (Fig. 5f, h) and the pygidial ectoderm (Fig. 5g) expands to encompass more cells, although there are still fewer *Ct*-*pros* ^+^ cells at the dorsal midline within the peristomium. Some animals also begin to express *Ct*-*pros* in a few lateral ectodermal cells just posterior to the peristomium (Fig. 5h, arrow).

At stage 5, *Ct*-*pros* continues to be expressed in the anterior ectoderm, brain, trunk ectoderm, and pygidial ectoderm (Fig. 5i–l). Additionally, at stage 5, *Ct*-*pros* is expressed in the developing VNC (Fig. 5k, l, black arrowheads). At stage 6, *Ct*-*pros* expression decreases to a subset of cells in anterior neuroectoderm and in the lateral and medial brain (Fig. 5m, n, q, r). Within the trunk at stage 6, *Ct*-*pros* is expressed in a subset of cells in the VNC (Fig. 5o, p, r, black arrowheads) and foregut (Fig. 5r) and in single cells throughout the ectoderm (Fig. 5o–q). Within the pygidium, *Ct*-*pros* is only expressed in the rectum (Fig. 5r, white arrowhead).

At stage 7, *Ct*-*pros* becomes further reduced in the episphere and is largely localized to the lateral sides of the brain (Additional file 6: Figure S6f, arrows). Within the trunk, *Ct*-*pros* is expressed in a subset of cells in the VNC (Additional file 6: Figure S6g, h, black arrowheads), in the foregut (data not shown), and in single cells in a circumferential band in the posterior ectoderm that encompasses the PGZ (Additional file 6: Figure S6g, bracket). Color product was also detected at the base of each chaeta at stage 7; however, this may be due to probe trapping rather than actual gene expression. Stage 8 and 9 animals also express *Ct*-*pros* along the lateral edges of the brain, in the pharynx and esophagus, in the VNC, and in the PGZ (Additional file 6: Figure S6i, j; black arrowheads denote expression in the VNC).

### *Ct*-*ngn* homolog expression

**Fig. 6:**
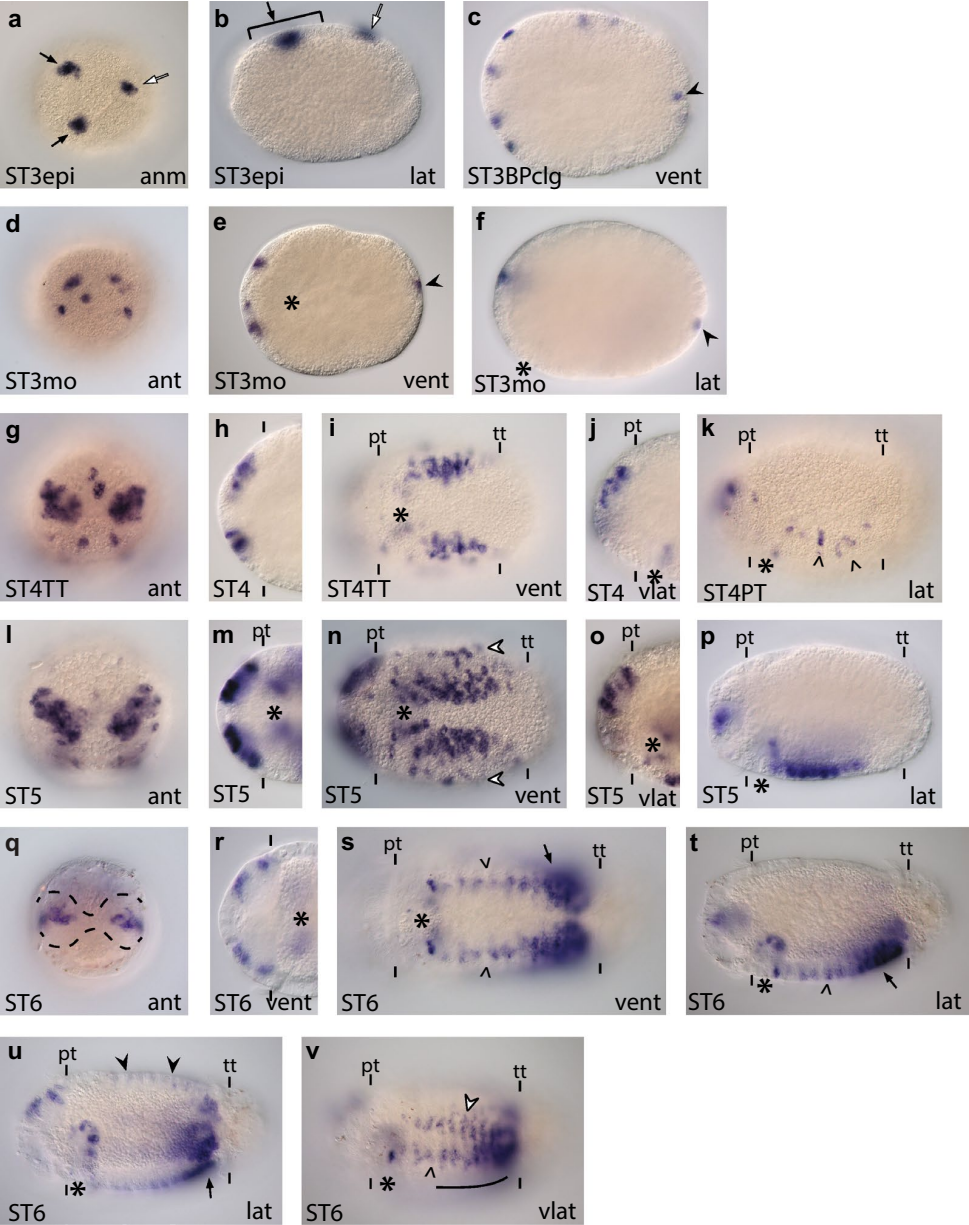
*Ct*-*ngn* is expressed in the neuroectoderm. **a**–**v** *Ct*-*ngn* transcripts were detected in stage 3–6 animals using WMISH. Images are to the same scale as in Figs. 2 and 3. In (**a**) and (**b**), *arrows* indicate *Ct*-*ngn* ^+^ presumptive neural cells, while the *white arrow* points to presumptive non-neural *Ct*-*ngn* ^+^ cells on the animal side of embryos undergoing epiboly. In (**b**), the *bracket* indicates the position of the anterior neuroectoderm. *Black arrowheads* in panels (**c**), (**e**), and (**f**) denote expression in the pygidium. *Arrowheads* in (**k**) point to expression in the ventral neuroectoderm. *White arrowheads* in (**n**) indicate the position of lateral rows of *Ct*-*ngn* ^+^ ectodermal cells. The *arrows* in panels (**s**), (**t**), and (**u**) point to posteriorly localized expression that encompasses the PGZ. The *arrowheads* in panels (**s**), (**t**), and (**v**) point to expression in the ventral neuroectoderm. *Black arrowheads* in (**u**) point to a row of *Ct*-*ngn* ^+^ dorsolateral ectodermal cells. The *white arrowhead* in (**v**) points to expression in the ventrolateral ectoderm, while the *line* denotes the position of the ventral midline. In each panel, the stage of the animal is indicated in the *lower left*, and the view is indicated in the *lower right* (*lat* lateral, *vlat* ventrolateral, *vent* ventral, *ant* anterior). In all lateral and ventrolateral views, anterior is to the left and ventral down; in all ventral views, anterior is to the left; in all anterior views, ventral is down. Panels (**h**), (**m**), and (**r**) are cropped images of the episphere from a ventral view. Panels (**j**) and (**o**) are cropped images of the episphere from a ventrolateral view. An *asterisk* marks the position of the mouth, and the prototroch (pt) and telotroch (tt) are labeled with *dashes*. In panels (**h**) and (**r**), *dashes* mark the prototroch. ST3epi, stage 3 epiboly; STBPclg, stage 3 blastopore closing; ST3mo, stage 3 mouth

Neurogenin (Ngn) homologs belong to the bHLH group A family of transcription factors and are involved in neuronal differentiation and specification of neuronal subtypes [90]. We identified one *neurogenin* homolog in *C. teleta*, *Ct*-*ngn*. Based on previous gene orthology analyses carried out by Simionato et al., Ct-Ngn (Cc Neurogenin) clusters within the Neurogenin clade [48, 91]. We characterized gene expression from cleavage stages (data not shown) through larval stages (Fig. 6; Additional file 7: Figure S7a–d).

*Ct*-*ngn* transcript was detected in zygotes through early cleavage stages (two cells through birth of the third quartet; data not shown). In stage 3 embryos undergoing epiboly, *Ct*-*ngn* is expressed on the animal side of the embryo in three patches, each patch comprising one to two cells (Fig. 6a, b, arrows and white arrow). Expression was also detected in a few smaller posteriolateral cells (data not shown). Two of the three patches of *Ct*-*ngn* ^+^ cells appear to be at the lateral edges of the anterior ectodermal thickening (Fig. 6a, b, arrows; anterior ectodermal thickening is bracketed in b), while the other *Ct*-*ngn* ^+^ patch of expression does not appear to be within the anterior ectodermal thickening (Fig. 6a, b, white arrow). As the blastopore begins to close, three to five single, large cells (usually four) in the anterior ectodermal thickening and a few smaller cells in the lateral and pygidial ectoderm express *Ct*-*ngn* (Fig. 6c; black arrowhead marks pygidial expression). By the end of stage 3, once the mouth opening is apparent, *Ct*-*ngn* expression is localized to a several single cells in the anterior neuroectoderm and the pygidium (Fig. 6d–f; black arrowhead indicates pygidial cells). The expression of *Ct*-*ngn* is similar from late stage 3 to early stage 4, with the addition of a few *Ct*-*ngn* ^+^ cells in the ventral neuroectoderm (Fig. 6k, arrowheads), in the ectoderm just posterior to the prototroch, and around the stomodeum, likely in the presumptive foregut (data not shown). At the end of stage 4, *Ct*-*ngn* expression in the anterior and ventral neuroectoderm expands (Fig. 6g–j), and new expression in a few dorsal and ventral cells in the ectoderm of the episphere can be detected (Fig. 6g). Pygidial expression of *Ct*-*ngn* was not detected in late stage 4 larvae (data not shown).

By stage 5, increasing numbers of superficial cells in the anterior neuroectoderm express *Ct*-*ngn* (Fig. 6l, m, o). The trunk expression expands to a few cells in the dorsal ectoderm (data not shown), cells in the foregut (Fig. 6n–p), surface cells in the ventral neuroectoderm (Fig. 6n, p), and a ventrolateral row of ectodermal cells (Fig. 6n, white arrowheads). Expression in the ventral neuroectoderm is a salt-and-pepper pattern, with highly expressing and weakly expressing cells interspersed with cells that do have expression (Fig. 6n). By stage 6, *Ct*-*ngn* expression in the episphere begins to decrease and is localized to a small region of anterior neuroectoderm (Fig. 6q, r, t, u). In the ventral neuroectoderm, expression decreases from anterior to posterior during stage 6, similar to the description of *Ct*-*ash1* below. However, the decrease in *Ct*-*ngn* expression appears to precede that of *Ct*-*ash1* (data not shown). Within the ventral neuroectoderm, in the regions where *Ct*-*ngn* expression has decreased, expression remains present in two rows of superficial cells overlying the lateral edges of the VNC (Fig. 6s, t, v, arrowheads) and in a few superficial cells near the stomodeum (Fig. 6s, t). This contrasts with expression in the more posterior ventral neuroectoderm, which has a much broader expression domain (Fig. 6s, t, u, arrow). Furthermore, expression in a small subset of cells within the VNC was detected at stage 6, but only after allowing the color reaction to proceed for a longer time. *Ct*-*ngn* also continues to be expressed in two rows of ventrolateral, ectodermal cells (Fig. 6v, white arrowhead and data not shown) and in the foregut (Fig. 6t, u). *Ct*-*ngn* expression also was faintly detected in additional cells in the ventrolateral (data not shown) and dorsolateral ectoderm (Fig. 6u; black arrowheads point to faint expression in the dorsolateral ectoderm).

From stages 7–8, *Ct*-*ngn* expression decreases considerably in most of the nervous system and is localized to superficial cells overlying the most posterior-most three to four ganglia of the VNC and to the PGZ (Additional file 7: Figure S7a–c). Within the PGZ, single *Ct*-*ngn* ^+^ cells can be detected ventrally and laterally in the ectoderm (Additional file 7: Figure S7b inset, black arrowhead). Faint, diffuse staining was also detected throughout the brain and VNC at these stages, but it was not clear whether this was actual expression because it was not ‘cellular’ in appearance. At stage 7, *Ct*-*ngn* continues to be expressed in single cells in the foregut and is expressed in a population of endodermal and possibly mesodermal cells in the trunk (Additional file 7: Figure S7a, b). By stage 9, *Ct*-*ngn* expression was only detected in the PGZ (Additional file 7: Figure S7d).

### *Ct*-*ash1* homolog expression

**Fig. 7:**
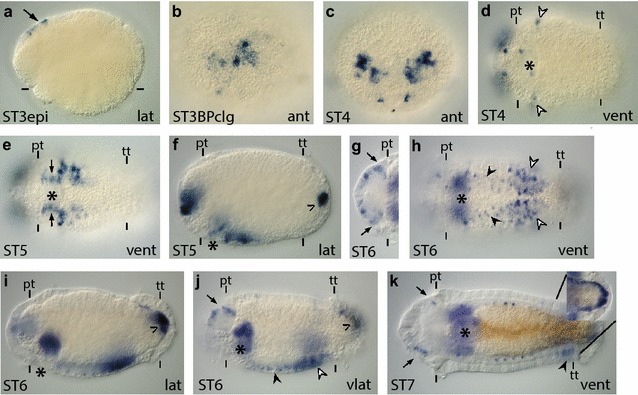
*Ct*-*ash1* is expressed in the neuroectoderm. (A–K) *Ct*-*ash1* transcripts were detected in stage 3–7 animals using WMISH. Images are to the same scale as in Fig. 2 and 3. *Arrow* in (**a**) indicates expression in the anterior neuroectoderm. The epibolizing front of the embryo is denoted with *dashes*. *White arrowheads* in (**d**) point to single cells expressing *Ct*-*ash1* in a circumferential ring posterior to the mouth opening. *Arrows* in (**e**) point to expression around the stomodeum. *Arrowheads* in panels (**f**), (**i**), and (**j**) denote expression in the presumptive visceral mesoderm. *Arrows* in panels (**g**), (**j**), and (**k**) point to expression at the lateral edges of the brain. In (**h**) and (**j**), *black arrowheads* point to a subset of expression in the anterior ventral neuroectoderm, and *white arrows* point to expression in the ventral neuroectoderm. *Black arrowhead* in (**k**) points to expression in the PGZ. In each panel, the stage of the animal is indicated in the *lower left*, and the view is indicated in the *lower right* (*lat* lateral, *vlat* ventrolateral, *vent* ventral, *dors* dorsal, *ant* anterior). In all lateral and ventrolateral views, anterior is to the left and ventral down; in all ventral views, anterior is to the left; in all anterior views, ventral is down. Panel (**g**) is a cropped image of the episphere from a ventral view. An *asterisk* marks the position of the mouth, and the prototroch (pt) and telotroch (tt) are labeled with *dashes*. ST3epi, stage 3 epiboly; STBPclg, stage 3 blastopore closing

Achaete–Scute complex homologs (Ash) belong to the bHLH group A family of transcription factors, similar to Ngn homologs [90, 92]. Five Achaete–Scute complex homologs were previously identified in *C. teleta*, two within the ASCa subgroup (Ct-Ash1 and Ct-Ash2) [50]. Expression of *Ct*-*ash1* during brain development was previously described [50]. In this article, we further characterize expression of *Ct*-*ash1* during VNC development (Fig. 7; Additional file 7: Figure S7a, b).

*Ct*-*ash1* transcript was detected from the four-cell stage through birth of the third quartet of micromeres (data not shown). We did not examine expression in zygotes or two-cell embryos. Expression of *Ct*-*ash1* in the brain from stages 3–6 has been previously described, so here we focus on expression in the trunk. To briefly summarize expression in the brain up to stage 6, it was found that *Ct*-*ash1* is expressed in patches of cells in the anterior neuroectoderm starting early during gastrulation (Fig. 7a, b) [50]. From stages 4–5, *Ct*-*ash1* continues to be expressed in superficial cells in the anterior neuroectoderm as well as in a subset of apically localized cells in the developing brain (Fig. 7c, f) [50]. By stage 6, *Ct*-*ash1* is largely excluded from the brain and is expressed in individual surface cells in the anterior neuroectoderm and at the lateral edges of the brain, which may represent regions of continuing NPC ingression (Fig. 7g, j arrows).

Within the trunk at stage 4, *Ct*-*ash1* transcripts are present in a few single cells around the stomodeum and in a circumferential ring of cells just posterior to the mouth opening (Fig. 7d, white arrowheads). By stage 5, *Ct*-*ash1* begins to be expressed in the anterior trunk in a salt-and-pepper pattern in the ventral neuroectoderm (Fig. 7e, f) and in a subset of cells in the developing VNC [50]. There is also expression in a cluster of cells in the pygidium (Fig. 7f, arrowhead). Expression around the stomodeum (Fig. 7e, arrows) and in the foregut also expands at this stage. By stage 6, more *Ct*-*ash1* ^+^ cells are detected in the foregut (Fig. 7h–j), ventral neuroectoderm (Fig. 7h, j, white arrows), which continues to have a salt-and-pepper pattern (Fig. 7h), and the VNC (Fig. 7h, j, black arrowheads). At the beginning of stage 6, *Ct*-*ash1* expression is localized to the anterior half of the neuroectoderm in the trunk (similar to Fig. 7e). As stage 6 progresses, this expression domain expands farther toward the posterior until it reaches the telotroch (data not shown). Then, toward the end of stage 6, the anterior-most limit of neuroectodermal expression in the trunk begins to regress posteriorly (Fig. 7h, i) such that expression appears to move from anterior to posterior. It was previously shown that neurons in the VNC form from anterior to posterior [52], and expression of *Ct*-*ash1* in the ventral neuroectoderm may prefigure this neuronal differentiation. In the pygidium, the domain of *Ct*-*ash1* expression expands anteriorly (Fig. 7i, j, arrowhead) [50], which may represent a subset of visceral mesodermal precursor cells.

At stage 7, *Ct*-*ash1* expression in the episphere is localized to a few, single cells located in the epidermis (Fig. 7k, arrows) and at the lateral edges of the brain (data not shown). Within the trunk at stage 7, *Ct*-*ash1* is restricted to the foregut, the presumptive posterior visceral mesoderm, and the PGZ, where neurogenesis is likely occurring (data not shown and Fig. 7k; black arrowhead points to PGZ and inset shows expression the presumptive visceral mesoderm). By stage 8, *Ct*-*ash1* was detected faintly in the brain, while in the trunk, it was found to be localized to the pharynx and ventral portion of the esophagus, posterior visceral mesodermal cells, and the PGZ (data not shown and Additional file 7: Figure S7e; black arrowhead points to PGZ). This expression pattern is largely the same at stage 9. (Additional file 7: Figure S7f). Expression in the visceral mesoderm at stage 9 is immediately adjacent to the midgut epithelium and extends from the posterior end of the larvae anteriorly to the esophagus (Additional file 7: Figure S7f, arrowheads).

### *Ct*-*neuroD* homolog expression

**Fig. 8:**
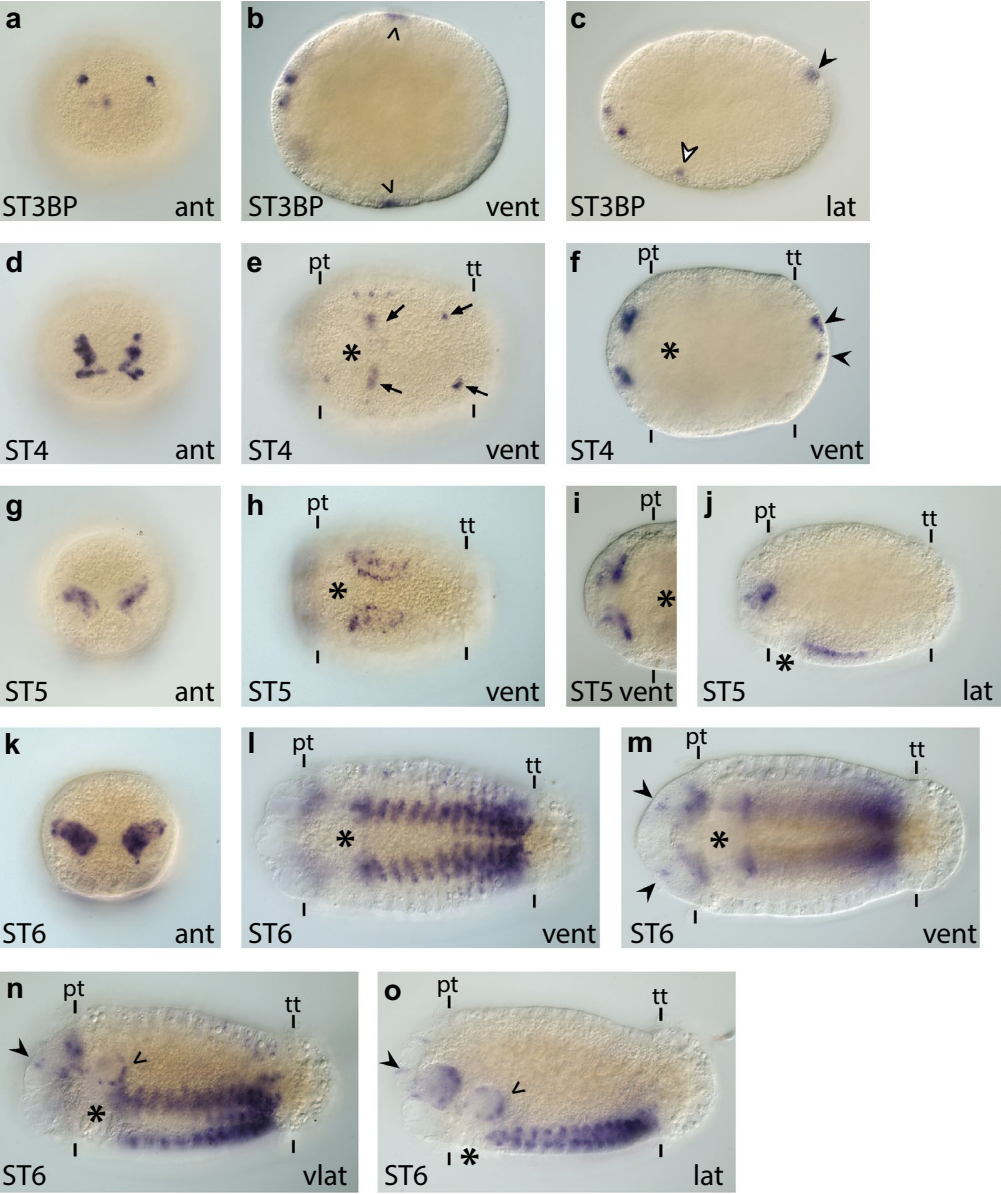
*Ct*-*neuroD* is expressed in subsurface cells within the developing CNS. **a**–**o** *Ct*-*neuroD* transcripts were detected in stage 3–6 animals using WMISH. Images are to the same scale as in Figs. 2 and 3. *Arrowheads* in (**b**) point to lateral patches of *Ct*-*neuroD* ^+^ cells in the trunk. *White arrowhead* in (**c**) points to a single *Ct*-*neuroD* ^+^ cell near the mouth opening. The *black arrowheads* in (**c**) and (**f**) indicate expression in the pygidium. *Arrows* in (**e**) point to a few *Ct*-*neuroD* ^+^ cells posterior to the mouth and in the ventral ectoderm. *Black arrowheads* in (**m**)–(**o**) indicate single *Ct*-*neuroD* ^+^ cells in the epidermis of the episphere. *Arrowheads* in panels (**n**) and (**o**) point to expression in the foregut. In each panel, the stage of the animal is indicated in the *lower left*, and the view is indicated in the *lower right* (*lat* lateral, *vlat* ventrolateral, *vent* ventral, *ant* anterior). In all lateral and ventrolateral views, anterior is to the left and ventral down; in all ventral views, anterior is to the left; in all anterior views, ventral is down. Panel (**i**) is a cropped view of the head. An *asterisk* marks the position of the mouth, and the prototroch (pt) and telotroch (tt) are labeled with *dashes*. STBP, stage 3 blastopore

NeuroD is a bHLH group A transcription factor that induces cell cycle exit and terminal differentiation in vertebrate embryos [90]. A NeuroD homolog has not been identified in the *D. melanogaster* genome [90, 91]. In *C. teleta*, we identified one homolog, *Ct*-*neuroD*. Gene orthology analyses carried out by Simionato et al. placed Ct-NeuroD (Cc NeuroD) within the NeuroD clade [48, 91]. We characterized *Ct*-*neuroD* expression during cleavage stages (none detected) and at embryonic and larval stages (Fig. 8; Additional file 7: Figure S7g–j).

*Ct*-*neuroD* transcripts were first detected during early epiboly, in a few cells on the animal side of the embryo (data not shown). Later during stage 3, once a blastopore becomes apparent, *Ct*-*neuroD* is expressed in a few (approximately three to five) small, single cells in the anterior neuroectoderm (Fig. 8a–c). At this stage, *Ct*-*neuroD* is also expressed in a few cells in the lateral ectoderm (Fig. 8b, arrowheads), two cells on either side of the presumptive stomodeum (Fig. 8c, white arrowhead), and one to two cells in the presumptive pygidium (Fig. 8c, black arrowhead). At stage 4, more subsurface cells in the anterior neuroectoderm (Fig. 8d, f) as well as a few cells in the ventral neuroectoderm (Fig. 8e, arrows), and the pygidium (Fig. 8f, black arrowheads) express *Ct*-*neuroD*.

At stage 5, as the brain lobes begin to be visible, *Ct*-*neuroD* expression is limited to subsurface cells in the forming brain (Fig. 8g, i, j). In the trunk, *Ct*-*neuroD* is present in the forming ganglia of the VNC (Fig. 8h, j). During stage 6, in the head, *Ct*-*neuroD* expression is localized to basal cells within the brain lobes (Fig. 8k, m, n, o), and a few cells in the epidermis overlying the brain (Fig. 8m–o, black arrowheads). Within the trunk at stage 6, *Ct*-*neuroD* expression is maintained in all of the forming ganglia in the VNC (Fig. 8l, n, o) and is visible in a few cells in the foregut (Fig. 8n, o, arrowheads).

At stages 7 and 8, expression was no longer detected in the brain (data not shown and Additional file 7: Figure S7h, i), and only a few *Ct*-*neuroD* ^+^ cells in the more mature VNC were identified (Additional file 7: Figure S7g, i, white arrowheads). *Ct*-*neuroD* is expressed throughout the recently formed, posterior-most ganglia (Additional file 7: Figure S7g–i). *Ct*-*neuroD* was detected in a few cells in the foregut and posteriorly localized cells at the interface between the mesoderm and endoderm (Additional file 7: Figure S7h, i; arrowheads point to the pharyngeal staining). In stage 9 larvae, *Ct*-*neuroD* was only detected in the posterior-most ganglion (Additional file 7: Figure S7j).

## Discussion

In this article, we characterize expression of the neurogenic homologs *Ct*-*soxB1*, *Ct*-*soxB*, *Ct*-*msi*, *Ct*-*pros*, *Ct*-*ash1*, *Ct*-*ngn*, and *Ct*-*neuroD* in the annelid *C. teleta*. Based on their spatiotemporal patterns of expression, it is likely that many of these genes are part of the neurogenic gene regulatory network that controls development of the CNS in *C. teleta*.

### Comparison among SoxB and bHLH group A family members in the developing CNS of *Capitella teleta*

*Ct*-*soxB1*, *Ct*-*soxB*, *Ct*-*ngn*, and *Ct*-*ash1* are all expressed very early in the anterior and ventral neuroectoderm and are the earliest genes in this study to be expressed in the anterior neuroectoderm. Because expression of these genes precedes ingression of brain NPCs [50], this suggests a possible role in early neurogenesis such as in the maintenance of brain NPCs. Interestingly, during epiboly, the pattern of *Ct*-*ngn* differs from the other three expressed genes. *Ct*-*ngn* is expressed in two patches, each consisting of one to two large cells, at the lateral edges of the anterior ectodermal thickening (Fig. 6a, b, arrows). In contrast, *Ct*-*soxB1*, *Ct*-*soxB*, and *Ct*-*ash1* are expressed in a medial patch of several cells (Fig. 2b, c, arrow; Fig. 3b, c, arrow; Fig. 7b). Another difference is that both *Ct*-*soxB1* and *Ct*-*soxB* are expressed in cells outside of the anterior neuroectoderm, unlike *Ct*-*ngn* and *Ct*-*ash1*, which appear to be restricted to the neuroectoderm. Once cells start to ingress, both *Ct*-*ngn* and *Ct*-*ash1* are expressed at varying levels in patches of surface cells, where cells divisions are occurring (see Fig. 7c, g as an example). *Ct*-*ngn* is largely restricted to surface cells, while *Ct*-*ash1* is also expressed in a few basally localized cells. This pattern is consistent with a proneural function for both genes (see below). *Ct*-*soxB* and *Ct*-*soxB1* continue to be expressed in neural and non-neural ectoderm in the episphere while NPCs are ingressing (i.e., at stage 4) and appear to be downregulated in the non-neural ectoderm beginning at stage 5, once fewer cells are ingressing (compare Fig. 2e with 2i). Interestingly, these genes are not restricted to the surface, where early neurogenic events occur. Instead, they are expressed throughout much of the developing brain, indicating that they may play multiple roles in neurogenesis, from NPC maintenance to neuronal commitment and specification of neuronal subtypes.

**Fig. 9:**
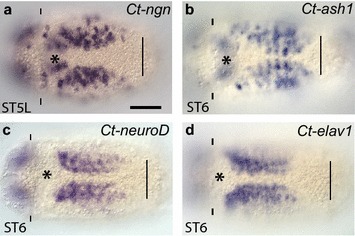
*Ct*-*neuroD* and *Ct*-*elav1* expression follows *Ct*-*ash1* and *Ct*-*ngn* during VNC development. **a**–**d** Comparison of the expression patterns of *Ct*-*ngn*, *Ct*-*ash1*, *Ct*-*neuroD*, and *Ct*-*elav1* in the developing VNC at late stage 5 (**a**) and stage 6 (**b**–**d**) as assayed by WMISH. The *scale bar* in A is 40 µm; all other images are to the same scale unless otherwise noted. In each panel, the images are shown in a ventral view, with anterior to the left, and the stage is indicated in the *lower left*. An *asterisk* marks the position of the mouth, the prototroch (pt) is labeled with *dashes*, and the position of the telotroch (tt) is marked by a *straight line*. ST5L, stage 5 late

In the trunk, *Ct*-*ngn* and *Ct*-*soxB1* are the earliest genes identified in this study to be expressed in the ventral neuroectoderm, at stage 4, followed shortly afterward by *Ct*-*ash1* and *Ct*-*soxB*, which both can be detected at stage 5 (compare Figs. 2g and 6i with Figs. 3i and 7e). By stage 6, both *sox* genes appear reduced in the ventral neuroectoderm and are present in the developing VNC, suggesting functions in NPCs early in CNS development and in maturing neurons later in CNS development. *Ct*-*ngn* and *Ct*-*ash1* are both expressed at varying levels in surface cells in the ventral neuroectoderm (Fig. 9a, b), where cells are actively dividing (NPM, unpublished observations). This combined with the anterior-to-posterior onset and downregulation of *Ct*-*ngn* and *Ct*-*ash1* expression in the ventral neuroectoderm suggests a function in maintaining NPCs and/or a proneural function. Because *Ct*-*ngn* is the only gene we identified that appears to be largely restricted to surface cells in the neuroectoderm, we think that this gene may function very early in neurogenesis, possibly in fate specification and/or maintenance of NPCs that generate the brain and VNC.

*Ct*-*neuroD* is also expressed early during gastrulation in the anterior neuroectoderm and slightly later in the ventral neuroectoderm; however, *Ct*-*neuroD* expression is entirely basal within the developing brain and VNC (e.g., Fig. 8f, j), similar to expression of the pan-neuronal genes *Ct*-*elav* and *Ct*-*syt1* [50, 52]. The onset of *Ct*-*neuroD* expression in the anterior and ventral neuroectoderm is similar to that of *Ct*-*ash1* and appears to precede *Ct*-*elav* and *Ct*-*syt1*. Within the developing VNC, the pattern of *Ct*-*neuroD* and *Ct*-*elav1* is very similar during stage 6 (Fig. 9c, d). These data suggest that *Ct*-*neuroD* functions in neural cells that have exited the cell cycle, and is consistent with our previous findings that there are few dividing subsurface cells [50].

**Fig. 10:**
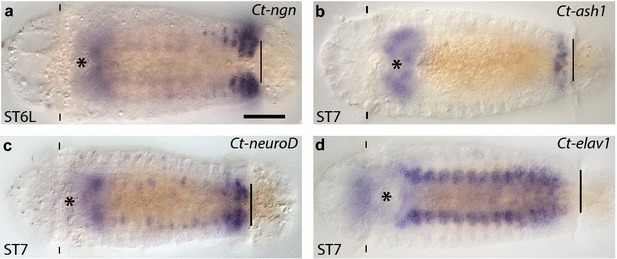
*Ct*-*ngn*, *Ct*-*ash1*, and *Ct*-*neuroD,* but not *Ct*-*elav1* expression terminate as the VNC matures. **a**–**d** Comparison of the expression patterns of *Ct*-*ngn*, *Ct*-*ash1*, *Ct*-*neuroD*, and *Ct*-*elav1* in the VNC at later stages of development. The *scale bar* in (**a**) is 40 µm; all other images are to the same scale unless otherwise noted. In each panel, the images are shown in a ventral view, with anterior to the left, and the stage of the animal is indicated in the *lower left*. An *asterisk* marks the position of the mouth, the prototroch (pt) is labeled with *dashes*, and the position of the telotroch (tt) is marked by a *straight . ST6L, stage 6 late*

As cell division and neurogenesis begins to be limited to the PGZ during stage 7, expression of the *Ct*-*soxB1*, *Ct*-*soxB*, *Ct*-*ngn*, and *Ct*-*ash1* becomes largely restricted to the PGZ (Fig. 10a–c). This is in contrast to *Ct*-*elav1* and *Ct*-*syt1*, which are absent from the PGZ, but continue to be expressed in the maturing ganglia throughout larval development (Fig. 10d and data not shown) [52]. The exceptions to this are that *Ct*-*soxB1*, *Ct*-*soxB*, *Ct*-*ngn*, and *Ct*-*neuroD* continue to be expressed in a small number of cells in the VNC. This spatiotemporal pattern of expression indicates that the *soxB* and bHLH homologs in *C. teleta* (*Ct*-*soxB1*, *Ct*-*soxB*, *Ct*-*ngn*, and *Ct*-*ash1*) play a prominent role in neurogenesis, similar to other bilaterian animals that have been analyzed. However, unlike the segregation of functions for SoxB and Ngn homologs in vertebrates, both *soxB* genes and *Ct*-*ngn* may function in dividing NPCs and differentiating neural cells in *C. teleta* (see below). Similar to many other bilaterian animals, *Ct*-*ash1* expression is consistent with promoting ingression and decreased proliferative capacity of NPCs while *Ct*-*neuroD* likely functions downstream of the other bHLH genes, in neuronal maturation.

### Comparison of *Ct*-*soxB1* and *Ct*-*soxB* expression patterns with other metazoans

In several vertebrates, SoxB1 homologs (Sox1, 2, and 3) are expressed in overlapping patterns in the developing CNS. Expression of these homologs begins prior to formation of the neuroectoderm and then becomes restricted to proliferating NPCs. As neural daughter cells begin to differentiate, expression of SoxB1 homologs becomes downregulated, although expression is maintained in a small subset of mature neurons in the CNS [12, 14–16, 20]. SoxB1 proteins (e.g., Sox2) function primarily in dividing NPCs, while SoxB2 proteins (e.g., Sox14 and 21) function in differentiating neural cells, and antagonize SoxB1 proteins [12, 13, 17, 18, 20, 22, 93–96]. In contrast to vertebrates, genome-wide transcription factor binding studies in *D. melanogaster* found that the SoxB proteins SoxNeuro and Dichaete function throughout neurogenesis, from neural fate specification to neuronal maturation, and likely upregulate many of the same target genes [26, 33].

*Capitella teleta Ct*-*soxB1* and *soxB* show similarities in expression with both insect and vertebrate homologs. Similar to vertebrates, *Ct*-*soxB1* and *Ct*-*soxB* are initially expressed more broadly in the ectoderm and then become restricted to the neuroectoderm, where NPCs are actively dividing. However, both *Ct*-*soxB1* and *Ct*-*soxB* also appear to play a role in maturing neurons, similar to insects, as expression in the brain and VNC is prominent. Furthermore, *Ct*-*soxB* expression largely overlaps that of *Ct*-*soxB1* in the developing CNS. One interpretation is that the two homologs may be similar to insects and have redundant neurogenic functions. Alternatively, the two SoxB homologs in *C. teleta* could have antagonistic functions, similar to vertebrate SoxB1 and SoxB2 proteins. Furthermore, based on co-expression with *Ct*-*ngn* and *Ct*-*ash1*, the Ct-SoxB proteins may not antagonize proneural proteins as they do in vertebrates, and may instead upregulate proneural gene expression as in *D. melanogaster*. Interestingly, in another annelid, *P. dumerilii*, at least one SoxB homolog has been identified, *Pdu*-*soxB*, and it is expressed early in the neuroectoderm with subsequent downregulation upon onset of proneural gene (*Pdu*-*neurogenin* and *Pdu*-*achaete*–*scute*) expression [48, 49]. This pattern of expression is similar to SoxB1 expression in vertebrates and contrasts with co-expression of the *soxB* and proneural genes in *C. teleta*. Finally, to gain a better understanding of the ancestral function of SoxB proteins in spiralians, it will be important to elucidate the neurogenic gene regulatory network in *C. teleta* for comparison with other spiralians.

Recent work examining expression and function of a SoxB ortholog, NvSoxB(2), in the cnidarian sea anemone *Nematostella vectensis* indicates a role in proliferating NPCs as well as in neural cells that are not dividing [97, 98]. NvSoxB(2)^+^ neural cells that are dividing co-express the bHLH gene *Nvath*-*like*, while those that have exited the cell cycle co-express the proneural gene *NvashA* [98]. This indicates that the interaction between SoxB homologs and bHLH type A family members in cnidarians may be more complex than a simple antagonistic relationship. Taken together, a picture is beginning to emerge in which SoxB homologs may have functioned at multiple stages of neurogenesis in the last common ancestor of cnidarians and bilaterians, a role that is supported by our data from *C. teleta*.

### Comparison of *Ct*-*ash1* and *Ct*-*ngn* expression patterns with other metazoans

Proneural bHLH transcription factors are key neurogenic factors in all bilaterian clades investigated so far [9]. Proneural genes are expressed and function during neurogenesis in cnidarians [99–105], indicating an ancestral function in this process. In contrast, in ctenophores, homologs of several genes controlling neuronal fate and patterning including Neurogenin, Achaete–Scute complex, and NeuroD are absent [106], making it difficult to infer what molecular mechanisms governed the progression from diffuse nervous systems to more centralized systems. In vertebrates, the traditional view of proneural proteins has been that they promote cell cycle exit, neuronal differentiation, cell migration, and specification of neuronal subtypes [9, 12, 13, 18–21, 31, 107, 108], which is similar to the proposed function of NvAshA in *N. vectensis*. In non-insect arthropods (e.g., chelicerates and myriapods), Achaete–Scute complex homologs are thought to promote neural differentiation since they are expressed in non-dividing neural cells [39–43, 109]. This is somewhat different from expression of proneural proteins in the proliferative NPCs of vertebrates and insects [9, 44, 45].

More recently, a genome-wide transcription factor binding study in mouse found that Ascl1 (Achaete–Scute-like 1) regulates genes involved in all phases of neurogenesis [108, 110, 111]. Furthermore, there was strong evidence that Ascl1 directly promotes proliferation in brain neural progenitors. Similar genome-wide studies have not yet been conducted for vertebrate Ngn homologs, making it unclear whether they also have the ability to promote cell proliferation [110]. Interestingly, in *D. melanogaster*, a genome-wide transcription factor binding study of Asense found dual functions in promoting proliferation of neuroblasts and differentiation of the ganglion mother cells [112].

In *C. teleta*, expression of *Ct*-*ngn* and *Ct*-*ash1* in surface cells in the anterior and ventral neuroectoderm is consistent with a function in proliferating NPCs. The ‘salt-and-pepper’ expression pattern of for both genes is very similar to that seen for other proneural genes in *D. melanogaster*, indicating that *Ct*-*ash1* ^+^ and *Ct*-*ngn* ^+^ cells may be undergoing lateral inhibition [9, 113]. However, whether the specification of these cells is mediated by Notch signaling still needs to be investigated. Furthermore, based on the early onset and pattern of *Ct*-*ngn* expression, we think that Ct-Ngn may play a similar role in promoting cell cycle progression as does mouse Ascl1 and insect Asense. In contrast, *Ct*-*ash1* expression begins slightly later than *Ct*-*ngn* in the neuroectoderm and is downregulated later in the ventral neuroectoderm than *Ct*-*ngn*, indicating that Ct-Ash1 could promote cell cycle exit. This segregation of functions could be similar to that proposed for *Nvath*-*like* (dividing NPCs) and *Nvash1* (differentiating neural cells) in *N. vectensis* [98, 104, 105]. However, preliminary experiments indicate that *Ct*-*ash1* is expressed in both EdU+and EdU− cells (NPM unpublished data). Similarly, in *P. dumerilii*, *Pdu*-*ash* and *Pdu*-*ngn* are expressed apically within the ventral neuroectoderm [48], in the same location as proliferating cells [114]. In addition, *Pdu*-*ngn* expression begins early in development, overlaps with *Pdu*-*soxB*, and has a widespread salt-and-pepper pattern throughout the anterior and ventral neuroectoderm, leading Simionato et al. to suggest that Pdu-Ngn could be the ‘major proneural’ factor in *Platynereis* [48]. This is similar to the expression pattern we have observed for *Ct*-*ngn*; however, *Ct*-*ngn* is also expressed later in a subset of cells in the VNC. *Pdu*-*ash* expression, while apically localized, is restricted to a subset of cells in the anterior and ventral neuroectoderm, suggesting that Pdu-Ash may specify neural subtypes [48]. In contrast, *Ct*-*ash1* is clearly expressed in both surface and subsurface cells.

### *Ct*-*neuroD* may function in neural differentiation in *C. teleta*

In mouse and *Xenopus laevis*, NeuroD homologs are expressed later than Ngn1 and 2 and primarily in post-mitotic neurons in the intermediate zone of the neural tube. Furthermore, NeuroD homologs are known to play a major role in neuronal differentiation and are direct transcriptional targets of Ngn 2 [31, 90, 107, 108, 115, 116]. NeuroD homologs are absent from *D. melanogaster* and *Ciona intestinalis* genomes, indicating lineage-specific losses [91]. In the annelids *P. dumerilii* and *C. teleta*, *Pdu*-*neuroD* and *Ct*-*neuroD* are expressed early in the neuroectoderm, unlike in vertebrates. However, *Ct*-*neuroD* is expressed in basally localized cells within the developing brain and VNC, while *Pdu*-*neuroD* is expressed more apically and overlaps with *Pdu*-*soxB* [48]. The expression of Ct-neuroD is consistent with a function in promoting neuronal differentiation. As NeuroD homologs have not been identified in early branching metazoans and are missing in certain taxa [91], the ancestral role of NeuroD is during bilaterian neurogenesis is unclear.

### *Ct*-*msi* functions in post-mitotic neural cells in *C. teleta*

Musashi homologs are RNA-binding proteins with functions in stem-cell self-renewal, NPC maintenance, and asymmetric cell divisions in mammals and *D. melanogaster* [73–76, 78, 79, 81, 117]. In *D. melanogaster*, Musashi is necessary for asymmetric division of sensory organ precursor (SOP) cells and differentiation of their neural daughters [75, 79, 80]. In addition, *msi* is localized to dividing neuroblasts during larval neurogenesis in *D. melanogaster* [79], and overexpression in the larval brain induced proliferation of undifferentiated neuroblasts [75]. The two Musashi paralogs in vertebrates, Msi-1 and Msi-2, are expressed in dividing NPCs [76, 78, 117–121], and in other chordates, Msi homologs are downregulated as the neural tube closes. Mouse Msi-2 is also widely expressed outside the CNS and in post-mitotic neurons.

Within spiralians, Msi homologs are expressed in the developing nervous system. In the planarian *Dugesia japonica*, expression of *msi* homologs is restricted to neuronal lineages, suggesting a role in neuronal differentiation [71]. In the annelid *P. dumerilii*, *Pdu*-*msi* is localized to the developing brain and VNC as well as the PGZ, where cells are actively dividing [122]. In *C. teleta*, *Ct*-*msi* was largely detected within the developing brain and VNC, and not in the overlying neuroectoderm, where most cell division occurs. Unlike in vertebrates, where *Msi*-*1* homologs are downregulated in differentiating neurons, in *C. teleta*, *Ct*-*msi* expression is largely restricted to cells contained within the developing brain and the VNC, where NPCs are undergoing differentiation. Furthermore, at later larval stages, once neurogenesis is likely complete in the brain and anterior regions of the VNC, *Ct*-*msi* continues to be broadly expressed in the brain and VNC and is absent from the PGZ. We interpret this expression to mean that Ct-Msi is not involved in NPC maintenance or in the earlier steps of neurogenesis.

### *Ct*-*pros* may be involved in asymmetric cell division of neural and non-neural ectodermal cells in *C. teleta*

Prospero (Pros) homologs encode homeodomain proteins that are predominantly expressed in neuronal lineages [83, 88, 123]. In both vertebrates and *D. melanogaster*, Prospero homologs regulate the balance between self-renewal and differentiation of neural stem cells [83, 84, 88, 112, 123–128]. In *D*. *melanogaster*, Prospero is pan-neuronal and the protein and mRNA are asymmetrically localized to the basal cortex of dividing neuroblasts and then to the basal GMC daughter cell after division. In GMC cells, Prospero enters the nucleus and induces cell cycle exit by antagonizing *asense* activity [83, 85, 86, 123, 127, 129, 130]. In the crustacean *Daphnia magna*, a *prospero* homolog was also found to be localized to the neuroblasts [87]. In the spider *C. salei*, clusters of cells expressing an *achaete*–*scute* homolog invaginate to form the VNC. These cells also express a *prospero* homolog throughout the process of invagination and neuronal differentiation [89].

In *C. teleta*, *Ct*-*pros* expression suggests a role in dividing NPCs, possibly in asymmetrically dividing cells. *Ct*-*pros* is detected in single, apical cells in the anterior and ventral neuroectoderm at similar stages as *Ct*-*ash1* and *Ct*-*ngn*. However, unlike *Ct*-*ash1* and *Ct*-*ngn*, *Ct*-*pros* also is expressed in many ectodermal cells outside of the anterior and ventral neuroectodermal domains, suggesting an additional function in non-neural ectoderm. *Ct*-*pros* also is found in a subset of cells within the brain and VNC, and expression in the VNC persists through stage 9, long after *Ct*-*ash1* and *Ct*-*ngn* are no longer expressed in the trunk. This suggests that *Ct*-*pros* may have additional functions in non-neural ectoderm and in neuronal subtypes. In contrast to the expression of *Ct*-*pros* in surface neuroectoderm, the *P. dumerilii* homolog, *Pdu*-*prox*, is expressed in groups of post-mitotic cells within the developing VNC, suggesting a function in differentiating neurons [49].

The expression of *Ct*-*pros* in single cells in the non-neural ectoderm of the episphere and trunk suggests that it may play a role in asymmetric division of these cells. Interestingly, one of these non-neural domains is the peristomium, where *Ct*-*pros* is detected in a circumferential band of ectodermal cells at stages 3–5 (Fig. 5d, f, k). The cells of the peristomium are morphologically distinct from other ectodermal cells in *C. teleta* [131], and to our knowledge, this is the first identified molecular marker that is predominantly expressed in the peristomial region of the trunk, if only for a short time. *Ct*-*pros* expression expands to include cells throughout the rest of the trunk ectoderm, which is most evident at stage 6 (Fig. 5o, p), indicating that the peristomial cells may initiate asymmetric cell division before the rest of the trunk. Alternatively, *Ct*-*pros* may function with other genes to specify the fate of cells in the peristomium.

## Conclusions

We have described the spatiotemporal expression patterns of genes in *C. teleta* whose homologs are known to have significant functions during neurogenesis across Metazoa. Based on the expression patterns reported here, we predict that all genes examined in this study are involved in a neurogenic gene regulatory network in *C*. *teleta*. *Ct*-*soxB1* and *Ct*-*soxB* are two of the earliest genes to be expressed, and their protein products may act to specify the neuroectoderm. Once the neuroectoderm has formed, we predict that Ct-SoxB1, Ct-SoxB, and Ct-Ngn function in dividing NPCs, possibly maintaining them in a proliferative state. Based on previous work [50], we think that it is likely that NPCs in *C. teleta* undergo asymmetric cell division in the surface neuroectoderm to generate daughters that will ingress and form the neurons of the brain and VNC. Ct-Pros may control this asymmetric cell division, while Ct-Ash1 may act in dividing NPCs to induce cell cycle exit and ingression. Ct-NeuroD likely functions in post-mitotic neural cells to direct neuronal differentiation. Finally, we think that all of the homologs examined in this study (Ct-SoxB1, Ct-SoxB, Ct-Ngn, Ct-Ash1, Ct-Pros, Ct-NeuroD, and Ct-Msi) play additional roles in post-mitotic neurons, although to varying degrees. Overall, spatiotemporal regulation of these genes in *C. teleta* has highlighted striking similarities as well as differences between certain aspects of neurogenesis as compared to other bilaterian clades, including variation even within annelids. Further validation of the function of these genes in *C. teleta* as well as continued comparative analyses is important to understand evolution of different nervous system architectures and their underlying developmental mechanisms. Our results add support to the idea of a common genetic toolkit controlling nervous system development in various cnidarian and bilaterian animals, although the molecular makeup of this toolkit has been rearranged to varying degrees during evolution, both within and across clades.

## Additional files

**Additional file 1: Figure 1.** Accession numbers for Sox family proteins used in the gene orthology analyses.

**Additional file 2: Figure 2.** Accession numbers for Msi family proteins used in the gene orthology analyses.

**Additional file 3: Figure 3.** Consensus tree for Sox family proteins. Bayesian tree produced from the alignment of Sox protein sequences produced as described in “Methods.” This topology represents the 50% majority-rule consensus tree resulting from 7500 trees generated. The posterior probability for each branch is indicated in black next to each node. For nodes also present in our maximum likelihood analysis (see “Methods”), the bootstrap support is indicated in blue beneath the posterior probability for that node. *Mus musculus* LEF1 is included as an outgroup sequence. Previously reported Sox family groups B-F are largely supported, with clear *Capitella teleta* orthologs (red). Taxa represented are: Apme, *Apis mellifera;* Bbe, *Branchiostoma belcheri;* Cte*, Capitella teleta;* Dme*, Drosophila melanogaster;* Lgi*, Lottia gigantea;* Mmu*, Mus musculus;* Sko*, Saccoglossus kowalevskii;* Smi*, Stegodyphus mimosarum;* Tca*, Tribolium castaneum*.

**Additional file 4: Figure 4.** *Ct*-*soxB1* and *Ct*-*soxB* are expressed in overlapping domains within the developing CNS. *Ct*-*soxB1* (a–d) and *Ct*-*soxB* (e–h) transcripts were detected at stages 7–9 using WMISH. Images are to the same scale as in Figs. 2 and 3. *Arrowhead* in (a) points to *Ct*-*soxB1* expression in the developing VNC. The *arrow* in (c) points to *Ct*-*soxB1* expression in the epidermis of the episphere. *Arrowheads* in (e) indicate *Ct*-*soxB* expression in the VNC. In each panel, the stage of the animal is indicated in the *lower left*, the view is indicated in the *lower right* (lat, lateral; vlat; ventrolateral; vent, ventral), and the gene name is indicated in the top right. In all lateral and ventrolateral views, anterior is to the left and ventral down; in all ventral views, anterior is to the left. An *asterisk* marks the position of the mouth, and the prototroch (pt) and telotroch (tt) are labeled with *dashes*.

**Additional file 5: Figure 5.** Consensus tree for Musashi family proteins. Bayesian tree produced from the alignment of Musashi protein sequences as described in “Methods.” This topology represents the 50% majority-rule consensus tree resulting from 7500 trees generated. The posterior probability for each branch is indicated in black next to each node. For nodes also present in our maximum likelihood analysis (see “Methods”), the bootstrap support is indicated in blue beneath the posterior probability for that node. Deleted in azoospermia-associated protein (Dazap) and heterogeneous nuclear ribonucleoprotein (Hnrp) sequences are included as outgroup sequences. A single Musashi clade is largely supported, with a clear *Capitella teleta* ortholog (red). Taxa represented are: Apme*, Apis mellifera;* Bbe*, Branchiostoma belcheri;* Bfl*, Branchiostoma floridae;* Crgi*, Crassostrea gigas;* Cte*, Capitella teleta;* Dama*, Daphnia magna;* Dja*, Dugesia japonica;* Dme*, Drosophila melanogaster;* Has*, Homo sapiens;* Ixri*, Ixodes ricinus;* Ixsc*, Ixodes scapularis;* Lgi*, Lottia gigantea;* Lipo*, Limulus Polyphemus;* Mmu*, Mus musculus;* Sko*, Saccoglossus kowalevskii;* Sme*, Schmidtea mediterranea;* Smi*, Stegodyphus mimosarum;* Stpu*, Strongylocentrotus purpuratus;* Tca*, Tribolium castaneum;* Teur*, Tetranychus urticae;* Xetr*, Xenopus tropicalis*.

**Additional file 6: Figure 6.** *Ct*-*msi* and *Ct*-*pros* are expressed in the brain and VNC during later stages of development. *Ct*-*msi* (a–e) and *Ct*-*pros* (**f**–**i**) transcripts were detected at stages 7–9 using WMISH. Images are to the same scale as in Figs. 2 and 3. *Arrows* in (f) point to *Ct*-*pros* ^+^ cells positioned at the lateral edges of the brain. The bracket in (g) indicates the posterior region of *Ct*-*pros* expression that includes the PGZ. *Black arrowheads* in **g**–**j** indicate *Ct*-*pros* expression in the VNC. In each panel, the stage of the animal is indicated in the *lower left*, the view is indicated in the *lower right* (lat, lateral; vent, ventral), and the gene name is indicated in the top right. In all lateral views, anterior is to the left and ventral down; in all ventral views, anterior is to the left. Panel (f) is a cropped view of the head. An *asterisk* marks the position of the mouth, and the prototroch (pt) and telotroch (tt) are labeled with *dashes*.

**Additional file 7: Figure 7.** *Ct*-*ash1*, *Ct*-*ngn*, and *Ct*-*neuroD* expression is restricted to the PGZ during later stages of development. *Ct*-*ngn* (a–d), *Ct*-*ash1* (e, f), and *Ct*-*neuroD* (g–j) transcripts were detected at stages 7–9 using WMISH. Images are to the same scale as in Figs. 2 and 3. The inset in (b) is a cropped view of the PGZ from the same animal from a different focal plane, and *Ct*-*ngn* expression in the PGZ is denoted with a *black arrowhead*. *Black arrowhead* in (e) denotes *Ct*-*ash1* expression in the PGZ. *Arrowheads* in (f) indicate expression of *Ct*-*ash1* in the presumptive visceral mesoderm. *White arrowheads* in panel (g) indicate *Ct*-*neuroD* ^+^ cells within the VNC. *Arrowheads* in panel (h) and (i) point to *Ct*-*neuroD* expression in the foregut. In each panel, the stage of the animal is indicated in the *lower left*, the view is indicated in the *lower right* (lat, lateral; vent, ventral), and the gene name is indicated in the top right. In all lateral and ventrolateral views, anterior is to the left and ventral down; in all ventral views, anterior is to the left. An *asterisk* marks the position of the mouth, and the prototroch (pt) and telotroch (tt) are labeled with *dashes*.
