# Supplementary figures and images for: Spatiotemporal regulation of nervous system development in the annelid Capitella teleta
Source: EvoDevo. 2017 Aug 1;8:13. doi: 10.1186/s13227-017-0076-8 (PMC5539756; doi:10.1186/s13227-017-0076-8)

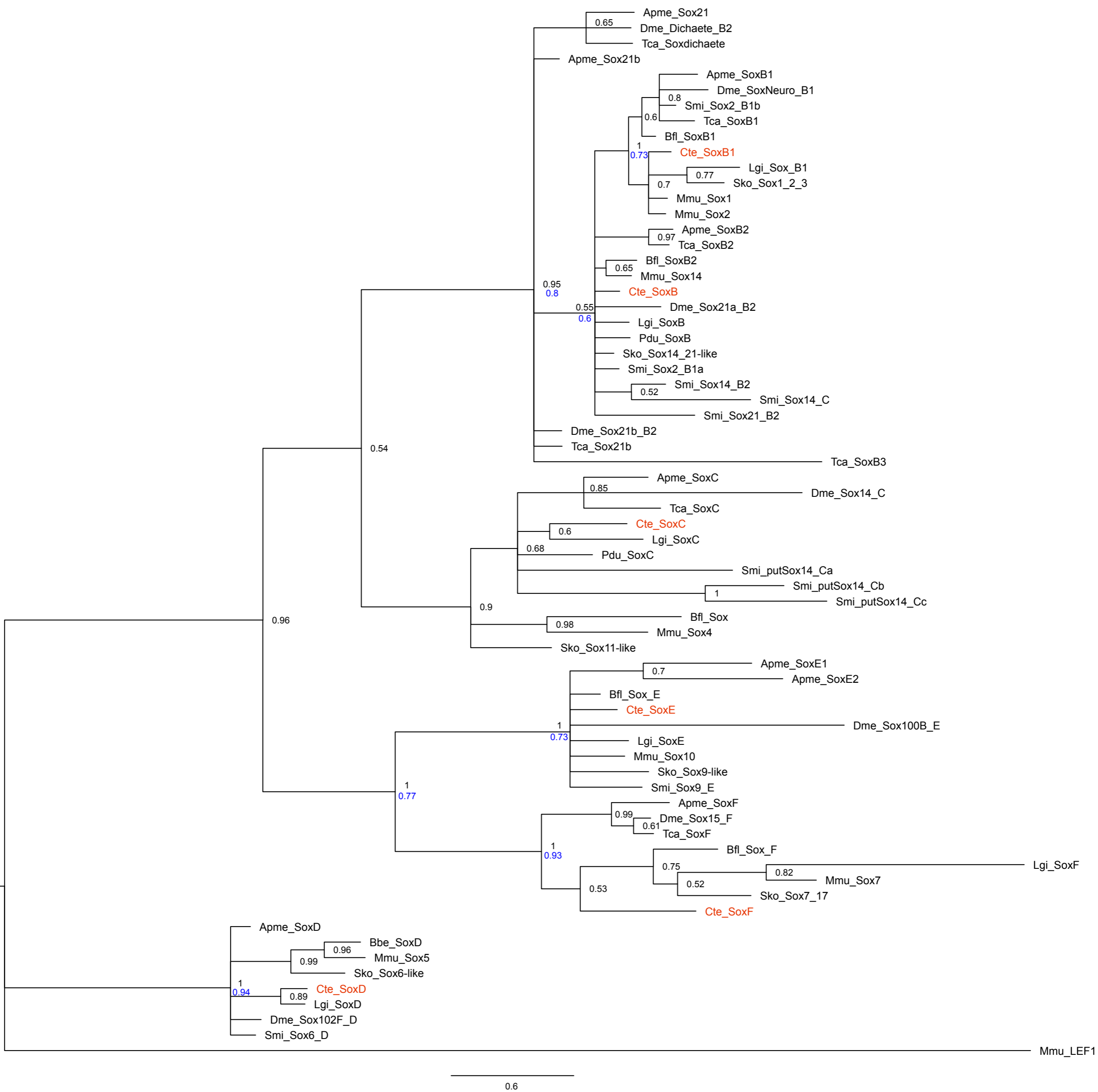

Supplement: Supplementary file 3 — Additional file 3: Figure 3. Consensus tree for Sox family proteins. Bayesian tree produced from the alignment of Sox protein sequences produced as described in “Methods.” This topology represents the 50% majority-rule consensus tree resulting from 7500 trees generated. The posterior probability for each branch is indicated in black next to each node. For nodes also present in our maximum likelihood analysis (see “Methods”), the bootstrap support is indicated in blue beneath the posterior probability for that node. Mus musculus LEF1 is included as an outgroup sequence. Previously reported Sox family groups B-F are largely supported, with clear Capitella teleta orthologs (red). Taxa represented are: Apme, Apis mellifera; Bbe, Branchiostoma belcheri; Cte, Capitella teleta; Dme, Drosophila melanogaster; Lgi, Lottia gigantea; Mmu, Mus musculus; Sko, Saccoglossus kowalevskii; Smi, Stegodyphus mimosarum; Tca, Tribolium castaneum. [file 13227_2017_76_MOESM3_ESM.pdf]

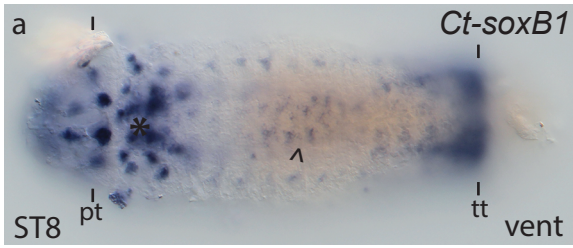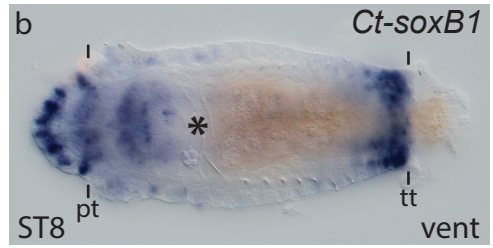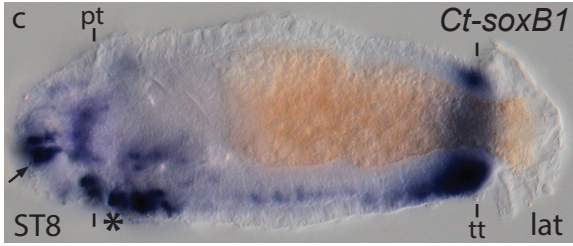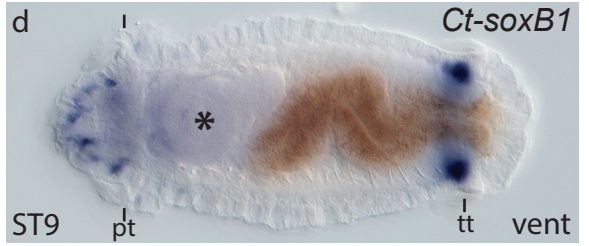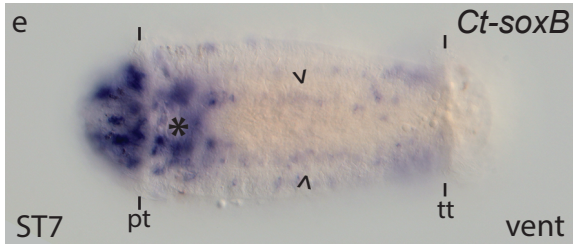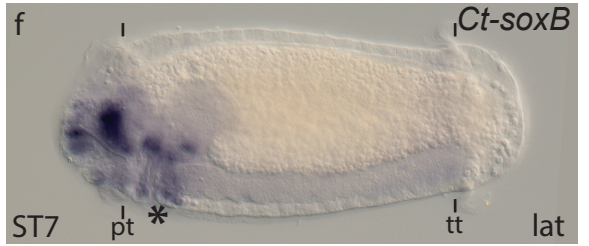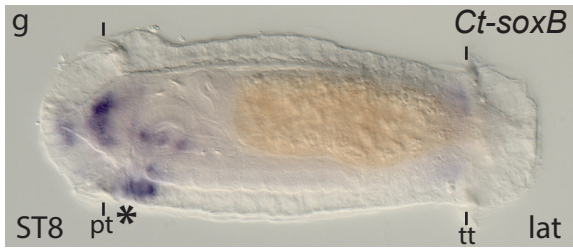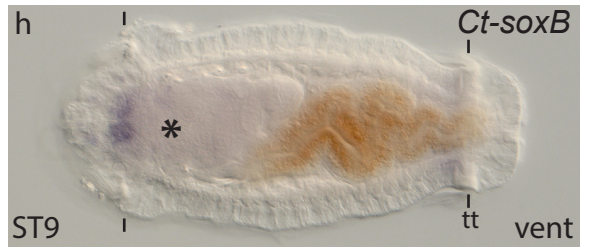

Supplement: Supplementary file 4 — Additional file 4: Figure 4. Ct-soxB1 and Ct-soxB are expressed in overlapping domains within the developing CNS. Ct-soxB1 (a–d) and Ct-soxB (e–h) transcripts were detected at stages 7–9 using WMISH. Images are to the same scale as in Figs. 2 and 3. Arrowhead in (a) points to Ct-soxB1 expression in the developing VNC. The arrow in (c) points to Ct-soxB1 expression in the epidermis of the episphere. Arrowheads in (e) indicate Ct-soxB expression in the VNC. In each panel, the stage of the animal is indicated in the lower left, the view is indicated in the lower right (lat, lateral; vlat; ventrolateral; vent, ventral), and the gene name is indicated in the top right. In all lateral and ventrolateral views, anterior is to the left and ventral down; in all ventral views, anterior is to the left. An asterisk marks the position of the mouth, and the prototroch (pt) and telotroch (tt) are labeled with dashes. [file 13227_2017_76_MOESM4_ESM.pdf]

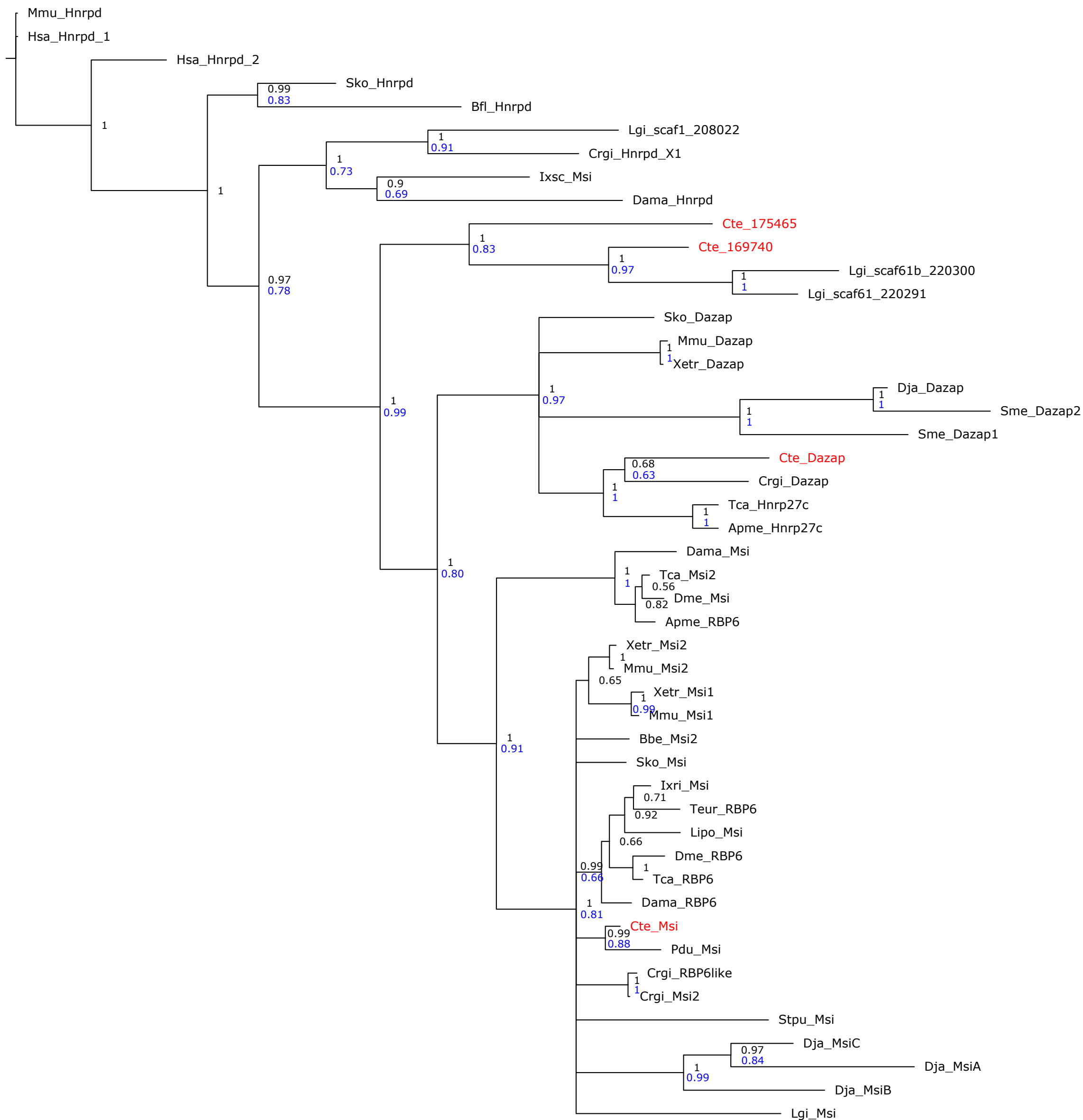

0.3

Supplement: Supplementary file 5 — Additional file 5: Figure 5. Consensus tree for Musashi family proteins. Bayesian tree produced from the alignment of Musashi protein sequences as described in “Methods.” This topology represents the 50% majority-rule consensus tree resulting from 7500 trees generated. The posterior probability for each branch is indicated in black next to each node. For nodes also present in our maximum likelihood analysis (see “Methods”), the bootstrap support is indicated in blue beneath the posterior probability for that node. Deleted in azoospermia-associated protein (Dazap) and heterogeneous nuclear ribonucleoprotein (Hnrp) sequences are included as outgroup sequences. A single Musashi clade is largely supported, with a clear Capitella teleta ortholog (red). Taxa represented are: Apme, Apis mellifera; Bbe, Branchiostoma belcheri; Bfl, Branchiostoma floridae; Crgi, Crassostrea gigas; Cte, Capitella teleta; Dama, Daphnia magna; Dja, Dugesia japonica; Dme, Drosophila melanogaster; Has, Homo sapiens; Ixri, Ixodes ricinus; Ixsc, Ixodes scapularis; Lgi, Lottia gigantea; Lipo, Limulus Polyphemus; Mmu, Mus musculus; Sko, Saccoglossus kowalevskii; Sme, Schmidtea mediterranea; Smi, Stegodyphus mimosarum; Stpu, Strongylocentrotus purpuratus; Tca, Tribolium castaneum; Teur, Tetranychus urticae; Xetr, Xenopus tropicalis. [file 13227_2017_76_MOESM5_ESM.pdf]

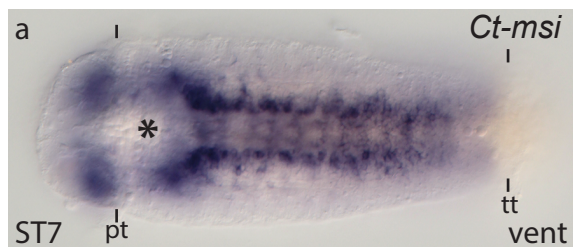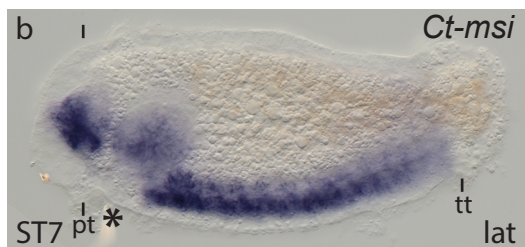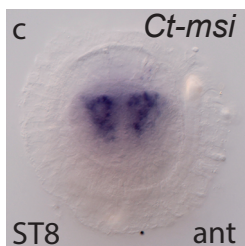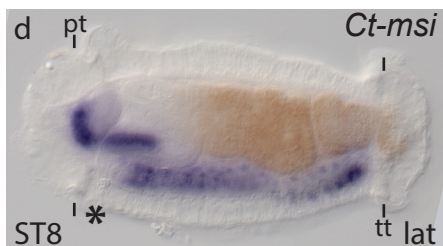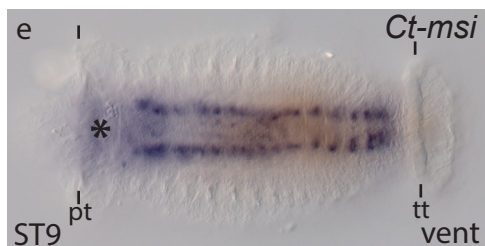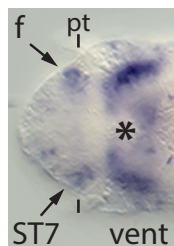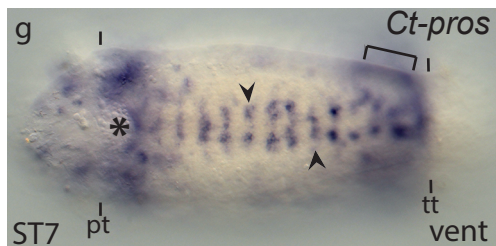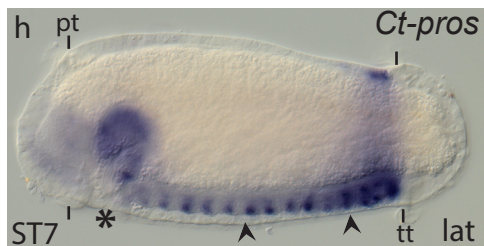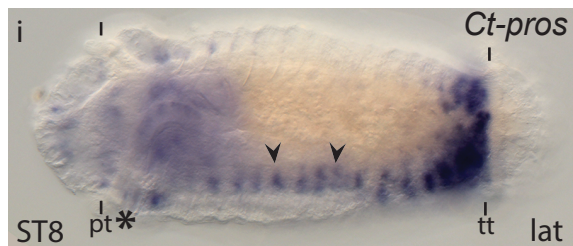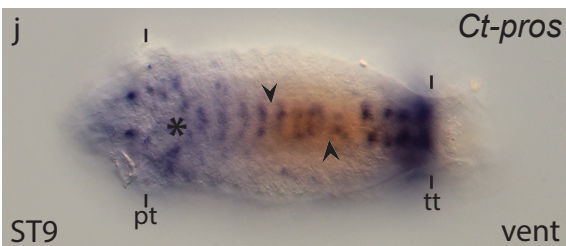

Supplement: Supplementary file 6 — Additional file 6: Figure 6. Ct-msi and Ct-pros are expressed in the brain and VNC during later stages of development. Ct-msi (a–e) and Ct-pros (f–i) transcripts were detected at stages 7–9 using WMISH. Images are to the same scale as in Figs. 2 and 3. Arrows in (f) point to Ct-pros + cells positioned at the lateral edges of the brain. The bracket in (g) indicates the posterior region of Ct-pros expression that includes the PGZ. Black arrowheads in g–j indicate Ct-pros expression in the VNC. In each panel, the stage of the animal is indicated in the lower left, the view is indicated in the lower right (lat, lateral; vent, ventral), and the gene name is indicated in the top right. In all lateral views, anterior is to the left and ventral down; in all ventral views, anterior is to the left. Panel (f) is a cropped view of the head. An asterisk marks the position of the mouth, and the prototroch (pt) and telotroch (tt) are labeled with dashes. [file 13227_2017_76_MOESM6_ESM.pdf]

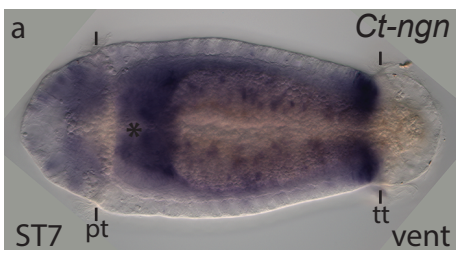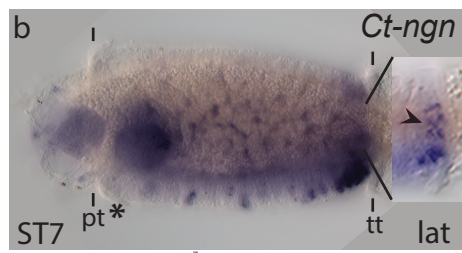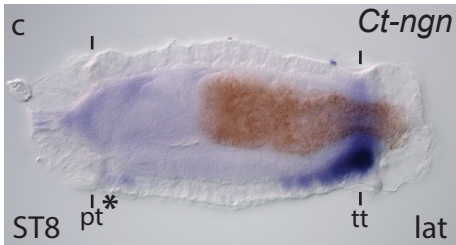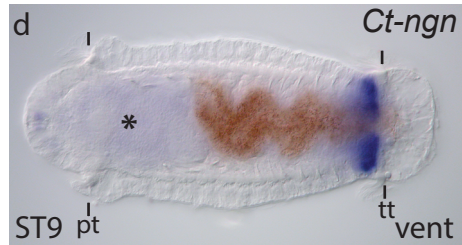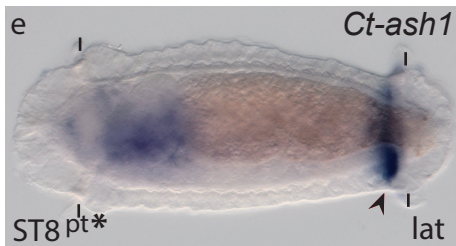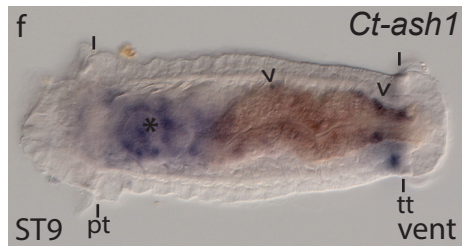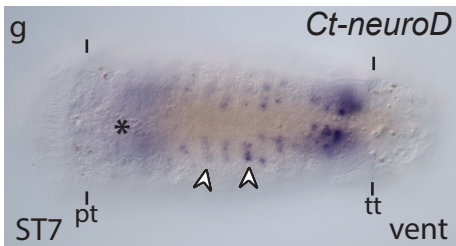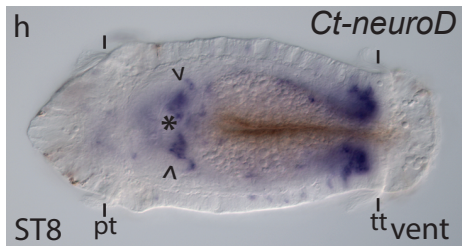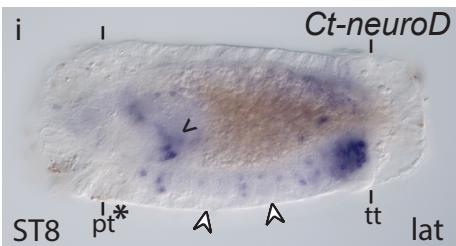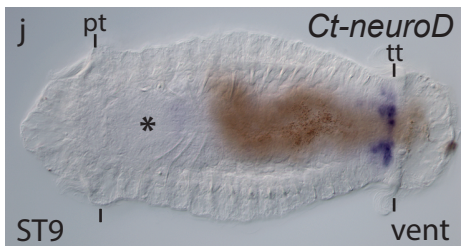

Supplement: Supplementary file 7 — Additional file 7: Figure 7. Ct-ash1, Ct-ngn, and Ct-neuroD expression is restricted to the PGZ during later stages of development. Ct-ngn (a–d), Ct-ash1 (e, f), and Ct-neuroD (g–j) transcripts were detected at stages 7–9 using WMISH. Images are to the same scale as in Figs. 2 and 3. The inset in (b) is a cropped view of the PGZ from the same animal from a different focal plane, and Ct-ngn expression in the PGZ is denoted with a black arrowhead. Black arrowhead in (e) denotes Ct-ash1 expression in the PGZ. Arrowheads in (f) indicate expression of Ct-ash1 in the presumptive visceral mesoderm. White arrowheads in panel (g) indicate Ct-neuroD + cells within the VNC. Arrowheads in panel (h) and (i) point to Ct-neuroD expression in the foregut. In each panel, the stage of the animal is indicated in the lower left, the view is indicated in the lower right (lat, lateral; vent, ventral), and the gene name is indicated in the top right. In all lateral and ventrolateral views, anterior is to the left and ventral down; in all ventral views, anterior is to the left. An asterisk marks the position of the mouth, and the prototroch (pt) and telotroch (tt) are labeled with dashes. [file 13227_2017_76_MOESM7_ESM.pdf]
